# Hypersensitive MR angiography based on interlocking stratagem for diagnosis of cardiac-cerebral vascular diseases

**DOI:** 10.1038/s41467-023-41783-9

**Published:** 2023-10-02

**Authors:** Peisen Zhang, Junwei Cheng, Yijie Lu, Ni Zhang, Xiaoai Wu, Hua Lin, Wei Li, Jian Wang, Mitchell A. Winnik, Zhihua Gan, Yi Hou

**Affiliations:** 1 College of Life Science and Technology, Beijing University of Chemical Technology, Beijing, 100029 China; 2 Department of Chemistry, University of Toronto, Toronto, ON M5S 3H6 Canada; 3 Department of Psychiatry, and Department of Nuclear Medicine, West China Hospital, Sichuan University, Chengdu, 610041 China; 4 Department of Nanomedicine & International Joint Cancer Institute, Naval Medical University, Shanghai, 200433 China; 5 Department of Head and Neck Surgery, National Cancer Center/National Clinical Research Center for Cancer/Cancer Hospital, Chinese Academy of Medical Sciences, Peking Union Medical College, Beijing, 100021 China

**Keywords:** Vascular diseases, Polymers, Cardiology, Translational research, Nanoparticles

## Abstract

Magnetic resonance (MR) angiography is one of the main diagnostic approaches for cardiac-cerebral vascular diseases. Nevertheless, the non-contrast-enhanced MR angiography suffers from its intrinsic problems derived from the blood flow-dependency, while the clinical Gd-chelating contrast agents are limited by their rapid vascular extravasation. Herein, we report a hypersensitive MR angiography strategy based on interlocking stratagem of zwitterionic Gd-chelate contrast agents (PAA-Gd). The longitudinal molar relaxivity of PAA-Gd was 4.6-times higher than that of individual Gd-chelates as well as appropriate blood half-life (73.8 min) and low immunogenicity, enabling sophisticated micro-vessels angiography with a resolution at the order of hundred micrometers. A series of animal models of cardiac-cerebrovascular diseases have been built for imaging studies on a 7.0 T MRI scanner, while the clinical translation potential of PAA-Gd has been evaluated on swine on a 3.0 T clinical MRI scanner. The current studies offer a promising strategy for precise diagnosis of vascular diseases.

## Abstract (web summary)

Current contrast-enhanced magnetic resonance angiography approaches are sub-optimal. Here the authors present a hypersensitive MR angiography strategy based on interlocking stratagem of zwitterionic Gd-chelate contrast agents (PAA-Gd), enabling sophisticated micro-vessel angiography of cardiac-cerebrovascular diseases with ultrahigh resolution.

## Introduction

Cardiovascular and cerebrovascular diseases have become urgent threats to public health, and more than one-third of deaths worldwide are caused by cardiac-cerebral vascular diseases, including coronary heart disease, acute ischemic stroke, thrombus, etc^1–3^. Tremendous efforts have been made for the diagnosis and prognosis of cardiac-cerebral vascular diseases. High-resolution and sensitive angiography can provide visualized information on vascular abnormalities, and is crucial for the early prevention and effective treatment of vascular-related diseases^4–6^. In clinic, digital subtraction angiography (DSA) is considered the reference standard for the diagnosis of vascular diseases, although it is an invasive intervention tool^7,8^. Computed tomography angiography (CTA) and computed tomography perfusion (CTP) as easy-to-use imaging modalities have been widely applied in revealing the anatomical and functional information on vasculature. However, CTA and CTP require patients to be exposed to X-ray, and considerations for medical conditions are required for patients receiving iodinated contrast media to prevent allergic reactions^9,10^.

Owing to the high-spatial-resolution and unlimited tissue penetration depth, magnetic resonance imaging (MRI) has become an indispensable clinical modality in visualization of vascular abnormalities with its noninvasive nature, freedom from ionizing radiation, and wide choices of specific sequences developed for MR angiography to improve image quality^11–13^. For instance, Time-of-flight (TOF) and phase-contrast (PC) MR angiography can provide angiograms with high spatiotemporal resolution based on blood flow^14,15^. However, these non-contrast-enhanced sequences suffer from their intrinsic problems, i.e., the respiratory artifact caused by the long image acquisition times; blood flow-dependency make it difficult to attain the required accuracy, especially for the vessels with complex structure or slow blood flow^16,17^. Therefore, it is pivotal to introduce exogenic contrast agents to enhance the anatomical structure and distribution of vasculature.

To date, numerous MRI contrast agents have been developed for precise medical diagnosis. For example, the paramagnetic Gd-chelates dominate the *T*_1_-weighted contrast agents in clinical practice, meanwhile the superparamagnetic iron oxide nanoparticles have been approved by Food and Drug Administration (FDA) as *T*_2_-weighted contrast agents^18^. Compared to *T*_2_-weighted contrast agents, the Gd-chelate derivants, such as Gd-DTPA, can shorten the *T*_1_ relaxation time of protons to enhance the brightness of blood, and thus have been widely applied in clinical MRI diagnosis of vasculopathy^19,20^. However, some disadvantages of the small molecular Gd-chelate contrast agents hinder their clinical applications, particularly in angiography. Firstly, owing to the small hydrodynamic sizes (e.g., ~0.18 nm for Gd-DTPA), the existing clinical MRI contrast agents exhibit short tumbling time (*τ*_R_), which influences the spin-lattice relaxation of water protons with Gd^3+^, leading to relatively low longitudinal molar relaxivity *r*_1_^21^. Additionally, the small molecular Gd-chelates can extravasate from blood vessels very soon, resulting in a very short blood circulation time, thus the MR angiography with high resolution, especially for the tiny blood vessels, is still confronted with great challenge^22–24^. Meanwhile, the small molecular Gd-chelate contrast agents can rapidly cross the capillary wall and then distribute themselves in the extracellular space of the tissues, which increases the overall signal of the organs but reduces the signal-to-background ratio of angiography^25,26^.

To address these restrictions, nanoparticle-based MRI contrast agents, such as Gd-doped inorganic nanoparticles, have been developed to improve the accuracy of MR angiography^27^. Compared to small molecular Gd-chelates, the Gd-doped nanoparticles have much longer *τ*_R_, and thus their *r*_1_ can be improved to 2~3-fold higher than that of Gd-chelates^28^. In addition, the blood circulation time of nanoparticle-based contrast agents can be improved greatly through biocompatible surface engineering, including PEGylated modification and protein coating^29,30^. Besides Gd-doped nanoparticles, *T*_1_-weighted MRI contrast agents based on iron oxide nanoclusters^31^ and supramolecular amorphous-like iron oxide^32^ have also been developed for high-resolution MR angiography, respectively. Nevertheless, the immune clearance of hard nanoparticle-based contrast agents by reticuloendothelial system (RES) is significant, leading to long-term accumulation of nanoparticles in the liver and spleen, whose biological effects are still unclear^33–36^.

**Fig. 1:**
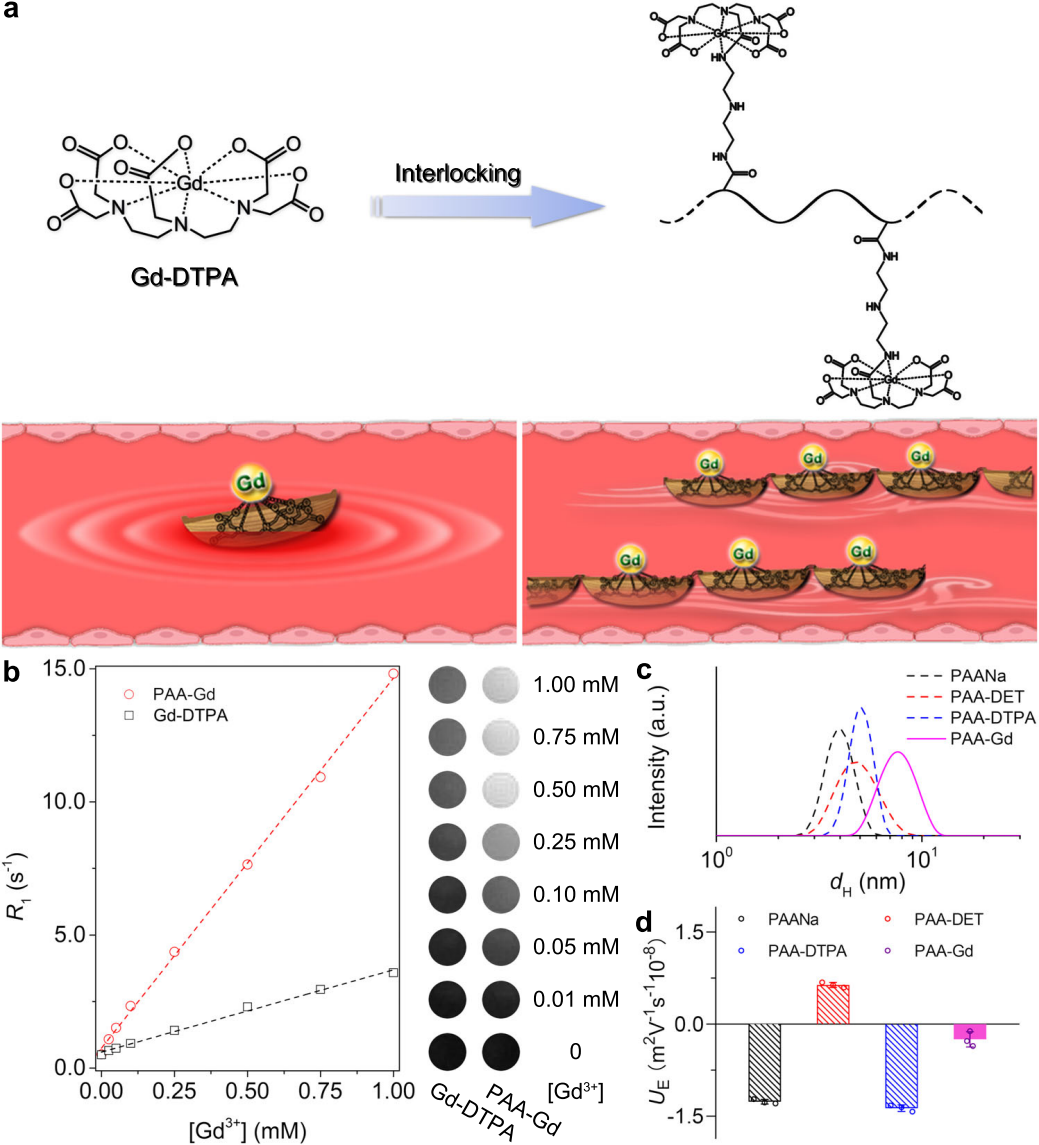
Illustration of interlocking stratagem and characterization of PAA-Gd. **a** The schematic illustration of zwitterionic Gd-chelate MR contrast agents compared with individual Gd-DTPA. **b** The linear regression fitting of the experimental data for extracting the longitudinal relaxivity *r*_1_, together with *T*_1_-weighted MR images of a series of aqueous solutions of PAA-Gd and Gd-DTPA, respectively. **c**, **d** The hydrodynamic size and electrophoretic mobility (*n* = 3, Data were plotted as mean ± standard deviation) of PAA, PAA-DET, PAA-DTPA, and PAA-Gd in 1× PBS (pH 7.4), respectively.

Inspired by the classical legend of interlocking stratagem, herein we developed a strategy of hypersensitive MR angiography with high resolution, based on a zwitterionic polymeric contrast agent. As illustrated in Fig. 1a, in order to restrict the tumbling of magnetic ions in blood stream, a polymer-carriers of magnetic ions were synthesized by coupling the diethylenetriaminepentaacetic acid (DTPA) molecules to poly(acrylic acid) (PAA) through a linker of diethylenetriamine according to our previous protocol^25^. The zwitterionic paramagnetic metal-chelating polymer (MCP) was subsequently obtained by chelating Gd^3+^ through DTPA groups, which is noted as PAA-Gd. The details of the synthesis and characterizations of PAA-Gd are provided in Supplementary Information. Due to the confined effect of polymer chain, the movement of single Gd-DTPA section is restricted, which dramatically slows down the tumbling rate of Gd^3+^. As a result, the *r*_1_ of PAA-Gd is 13.9 mM^−1^ s^−1^ evaluated by a clinic 1.5 T MRI scanner, while is *ca*. 4.6-fold higher than that of individual Gd-DTPA (3.0 mM^−1^ s^−1^), thus, its enhancement effect on *T*_1_-weighted MRI contrast is much more significant (Fig. 1b). Simultaneously, the hydrodynamic diameter (*d*_H_) of PAA-Gd is *~*7.7 nm, and its electrophoretic mobility is *ca*. −0.24 m^2^V^−1^s^−1^10^−^^8^ in physiological PBS buffer (pH 7.4) (Fig. 1c, d), namely, the contrast agent is almost neutral in physiological conditions. The ideal hydrodynamic size and zwitterionic structure could endow PAA-Gd with appropriate blood half-life and low immunogenicity. A series of animal models of cardiovascular and cerebrovascular diseases have been built for imaging studies on a 7.0 T animal MRI scanner with a three-dimensional dynamic contrast enhancement (3D DCE) sequence. Furthermore, the translation potential of PAA-Gd to the clinical settings has been evaluated through MR angiography of swine on a 3.0 T human MRI scanner.

## Results

### Fine vessel structures in head and thorax defined through 3D MR angiography

**Fig. 2:**
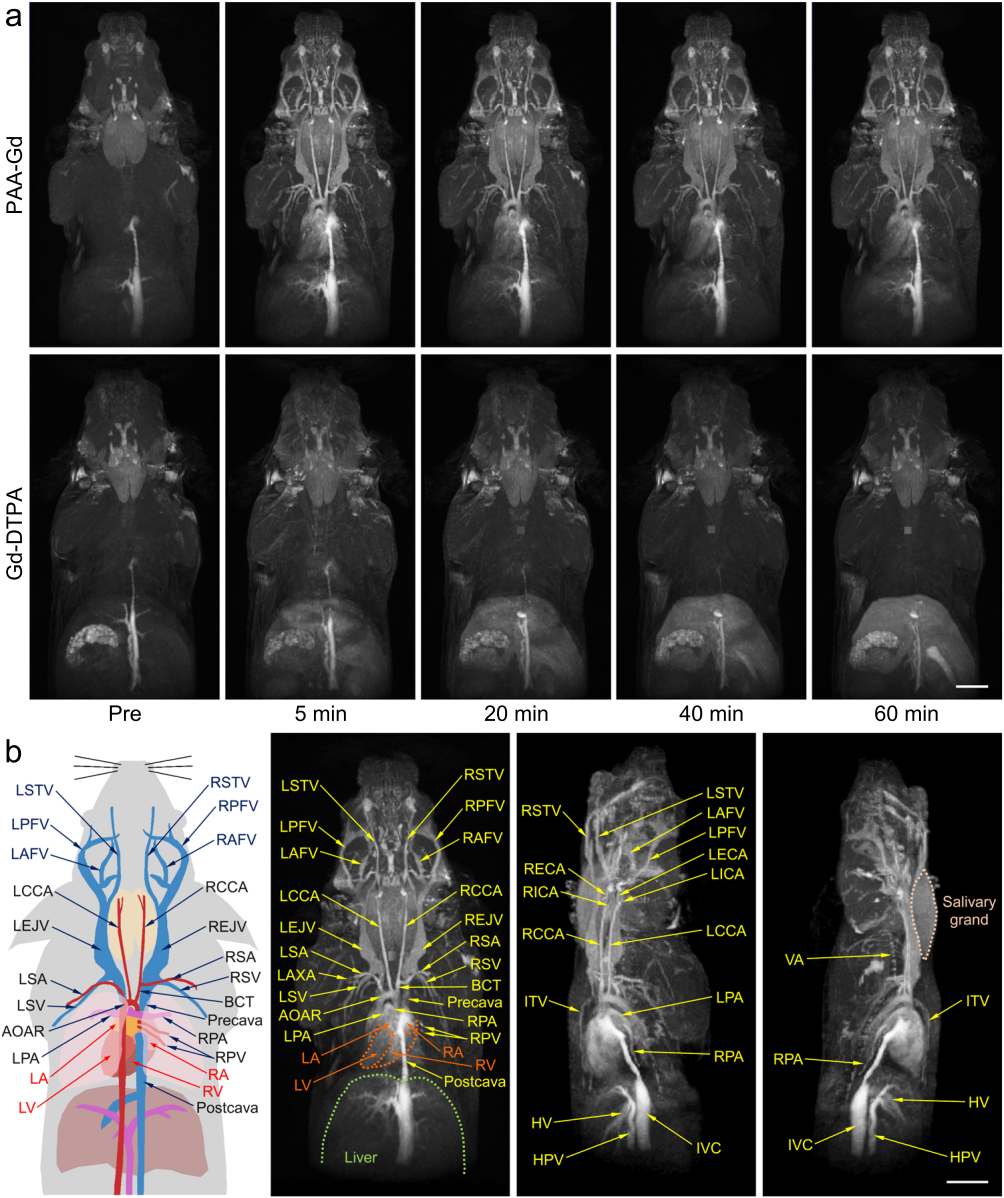
Head and thorax 3D MR angiography of mice with PAA-Gd. **a** Representative MR angiography obtained pre- and at different time points post-injection of PAA-Gd and Gd-DTPA. **b** Schematic drawing of vascular anatomy and the vascular identification in different directions of 3D angiography. Triplicates were performed independently with similar results. The embedded scale bar of frame **a** and **b** corresponded to 5 mm. Abbreviation: L/RSTV left/right superficial temporal vein, L/RPFV left/right posterior facial vein, L/RAFV left/right anterior facial vein, L/RECA left/right external carotid artery, L/RICA left/right internal carotid artery, L/RCCA left/right common carotid artery, L/REJV left/right external jugular vein, VA vertebral artery, L/RSA left/right subclavian artery, L/RSV left/right subclavian vein, BCT brachiocephalic trunk, AOAR, aortic arch, ITV internal thoracic vein, L/RPA left/right pulmonary artery, RPV right pulmonary vein, IVC inferior vena cava, HV hepatic vein, HPV hepatic portal vein, L/RA left/right atrium, L/RV left/right ventricle.

The enhancement effect of PAA-Gd on MR angiography contrast is first evaluated through 3D DCE MR angiography of the head and thorax of healthy mice, and the clinical Gd-DTPA contrast agent was adopted as control. As shown in upper panel of Fig. 2a and Supplementary videos 1 and 2, PAA-Gd can quickly distribute over each vascular branch accompanying with the bloodstream after the intravenous injection, and enhance the contrast of vessels evidently. More importantly, this angiography is still clearly visible without significant fading at 1 hour after injection, indicating that almost no PAA-Gd leaks out of blood vessels. Therefore, the PAA-Gd contrast agents can provide a suitable window period for the multiple complete DCE sequence scanning.

In comparison with PAA-Gd, the blood vessel distributions of mice treated with Gd-DTPA were barely visible, whereas only the contrast of liver parenchyma increased significantly with time (lower panel of Fig. 2a, Supplementary video 3 and 4), indicating Gd-DTPA extravasates from hepatic blood vessels rapidly after injection, and distributed in the extracellular space, especially distributes in liver, leading to difficulty in characterizing the structure of vascular system. For quantitatively evaluating the performance of these two contrast agents, the temporal evolution of the average relative *T*_1_ MRI signal intensities of vessels and hepatic parenchymal region were analyzed (Figs. S5 and  S6). The quantitative analysis results further support that PAA-Gd has long temporal window of MR angiography scanning and negligible diffusion out of the liver vessels, endowing PAA-Gd with the ability to largely enhance the spatiotemporal resolution of MR angiography.

Based on the vascular anatomic atlases of mice, the fine vessel structures of mouse in the probe-enhanced MR angiography are carefully identified. The schematic drawing of blood vessels and the 3D angiography results in different directions were given in the left and right panels of Fig. 2b. Accordingly, a great range of blood vessels can be observed clearly in the angiography, including main arteries, veins, and even tiny vessels with submillimeter diameter. Every clinically important vessel, especially the vessels of cardiovascular systems, such as aortic arch (AOAR), common carotid artery (CCA), subclavian artery/vein (SA/V), and even vertebral artery (VA), can be readily identified. In addition, although the heart of mouse beats very fast (400–600 times/min^37^), four main cardiac chambers can also be clearly distinguished (the red dotted line of demarcation), highlighting the excellent spatial resolution of MR angiography enhanced by PAA-Gd. Therefore, PAA-Gd contrast agents undoubtedly brings a qualitative leap to MR angiography, especially for the cardiovascular angiography.

### 3D MR angiography of cephalic and cervical vessels

Owing to the complicated structure of cephalic and cervical vessels, the theranostics of cardiac-cerebral vascular diseases is difficult in clinical practice. In this context, high-resolution 3D MR angiography of cephalic and cervical vessels is of great significance, because it can largely improve the diagnostic accuracy of these diseases, and provide reliable guidance for the individualized treatment strategy to patients, especially for those who undergo invasive surgery and need to vascular reconstruction as soon as possible.

In this respect, the in vivo PAA-Gd enhanced 3D MR angiography of cephalic and cervical vessels of rats was conducted. Before PAA-Gd injection, the clinically used axial time-of-flight (TOF) angiography sequence was adopted to show the vascular structures of the rat head. As shown in Fig. S7 and Supplementary Video 5, a rough sketch of blood vessels can be depicted. The large vessels that are perpendicular to the imaging plane can be observed. However, the micro-blood vessels cannot be clearly identified in TOF image, especially the microvessels with slow blood flow or oriented along the image plane, become progressively saturated and invisible.

**Fig. 3:**
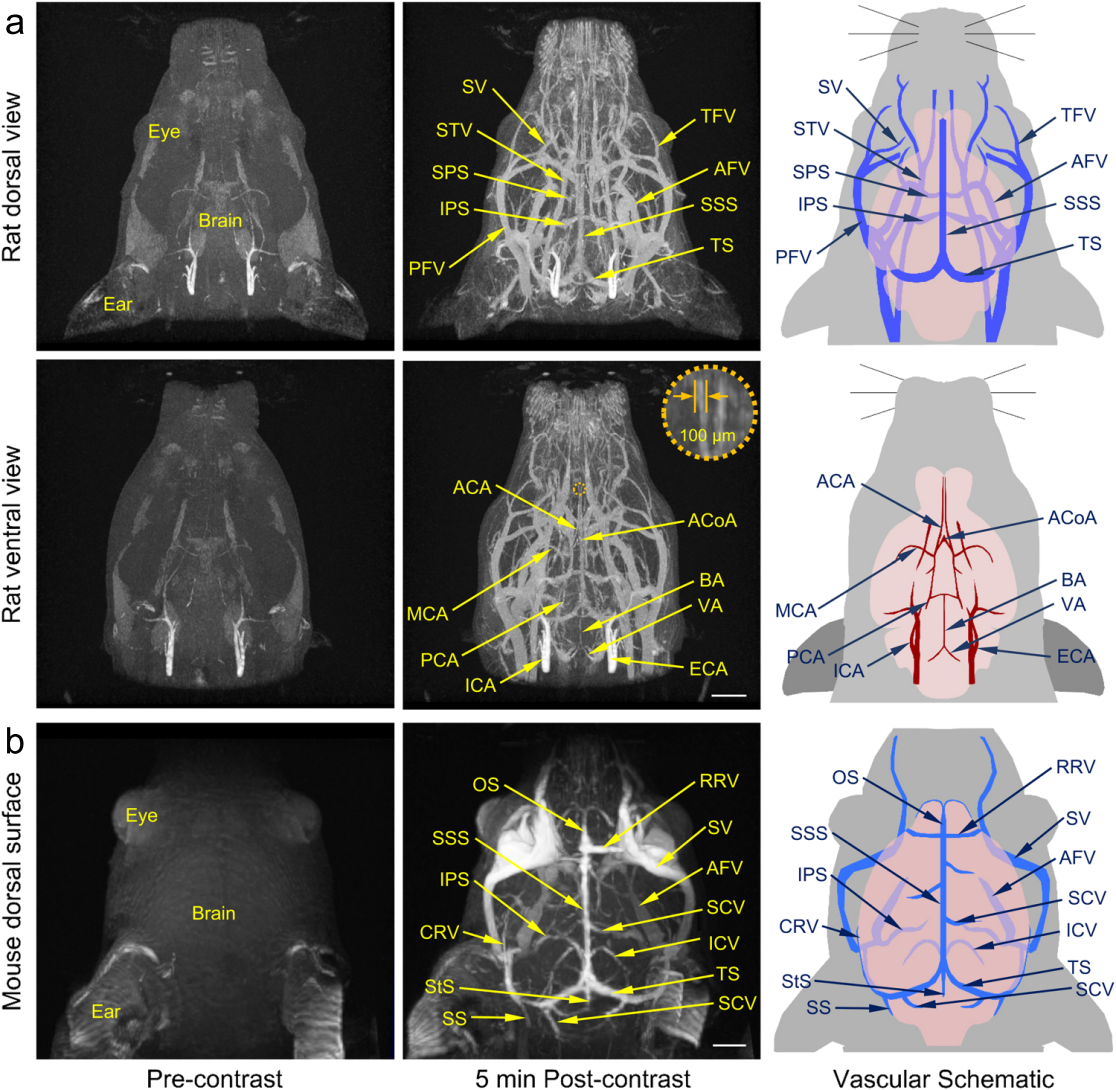
MR angiography of cerebrovascular with PAA-Gd. **a** The representative dorsal view (upper) and ventral view (lower) of rat head angiography obtained pre- and post-contrast of PAA-Gd through head coil, together with the schematic drawing of vascular anatomy (right penal). **b** The representative dorsal view of mouse head angiography obtained pre- and post-contrast of PAA-Gd through surface coil, together with the schematic drawing of vascular anatomy (right penal). The embedded scale bars of frame **a** and **b** corresponded to 5 mm and 2 mm, respectively. Triplicates were performed independently with similar results. Abbreviation: SV supraorbital vein, TFV transverse facial vein, STV superficial temporal vein, AFV anterior facial vein, PFV posterior facial vein, SPS superior petrosal sinus, IPS inferior petrosal sinus, SSS superior sagittal sinus, TS transverse sinus, ACA anterior cerebral artery, MCA middle cerebral artery, PCA posterior cerebral artery, AcoA anterior communicating artery, BA basilar artery, VA vertebral artery, ICA internal carotid artery, ECA external carotid artery, OS olfactory sinus, RRV rostral rhinal vein, SCV superior cerebral veins, ICV internal cerebral veins, CRV caudal rhinal vein, StS straight sinus, SS sigmoid sinus, SCV superficial cerebellar vein.

Thereafter, 3D MR angiography with PAA-Gd contrast agent was performed to identify the cerebrovascular structures. As shown in Fig. 3a and Supplementary Video 6 and 7, the sophisticated vessels in the rat brain, even as thin as 100 μm in diameter, were clearly visualized, whereas only the carotid artery can be vaguely observed without the contrast agent due to its fast blood flow velocity. Comparing with the illustration of artery and vein anatomy provided in the right panel of Fig. 3a, the important cephalic and cervical vessels, such as the basilar artery (BA), vertebral artery (VA), and transverse facial artery (TFV) can be clearly identified. More importantly, the tiny vessels of brain, including the superficial superior sagittal sinus (SSS), transverse sinus (TS) located, and the deep anterior/middle/posterior cerebral artery (A/M/PCA), which are closely related to cerebrovascular disease, were also distinguishable. Therefore, the PAA-Gd enhanced 3D MR angiography of cephalic and cervical vessels can serve as a rapid and reliable procedure for the precisely evaluation of arterial and venous changes.

For further evaluating the performance of PAA-Gd in cephalic and cervical vessel angiography, the right middle cerebral artery occlusion (rMCAO) was mechanically induced in rats to mimic the acute ischemic stroke. Approximately 24 hours after the model was established, the PAA-Gd enhanced 3D MR angiography of the rat head was conducted. As shown in Fig. S8, the blood structures of the head of stroke rat were clearly depicted. In comparison with the healthy rat, the blood signal of right carotid artery vanished, indicating the blockage of the blood vessel in rat head. To our surprise, the blood signal of the left carotid artery (red arrow) also reduced, indicating slowing down of the blood flow of this vessel. We speculated that the left carotid artery was probably compressed by inflammatory edema and swelling. More importantly, multiple tiny vessels can be observed surrounding the blocked arteries (yellow arrows), which implied that the compensatory angiogenesis occurred to restore the blood supply in ischemic region at 24 h post-modeling. These imaging results suggested that PAA-Gd enhanced 3D MR angiography can monitor the angiogenesis and the formation of tiny collateral vessels. Therefore, PAA-Gd-enhanced 3D MR angiography can visualize not only various vascular infarctions, but also abnormal blood vessel structures and angiogenesis after invasive surgery. These advantages will benefit the clinical diagnosis and evaluation of vascular injury and reconstruction.

Based on the above excellent imaging results, the angiography of cerebral cortical microvessels in rodent animal was further performed. The surface coil of mouse was employed for better displaying the superficial vascular structures. As shown in Fig. 3b, the sophisticated cerebral cortical microvessels and superficial tiny brain vessels of mouse were depicted by PAA-Gd contrast agent. Comparing with angiography carried out by conventional head coil (Fig. S9) that is not sensitive to the superficial vessels of the mouse head and mainly shows the internal vascular structures of the head including both the deep arteries and veins, the cerebral cortical microvessels including superior sagittal sinus (SSS), transverse sinus (TS), rostral rhinal vein (RRV), and olfactory sinus (OS) can be clearly displayed through the surface coil with higher resolution. Therefore, the vascular structure information obtained from these two coils is complementary, and both the two angiograms are of interest. Beneficial from the long blood half-life of PAA-Gd, it is possible to successively depict the superficial and internal vascular structures with enhanced 3D MR angiography following one single dose of PAA-Gd, because there will be a long enough imaging time window to alternately use different coils.

### 3D MR angiography of hepatic vessels

**Fig. 4:**
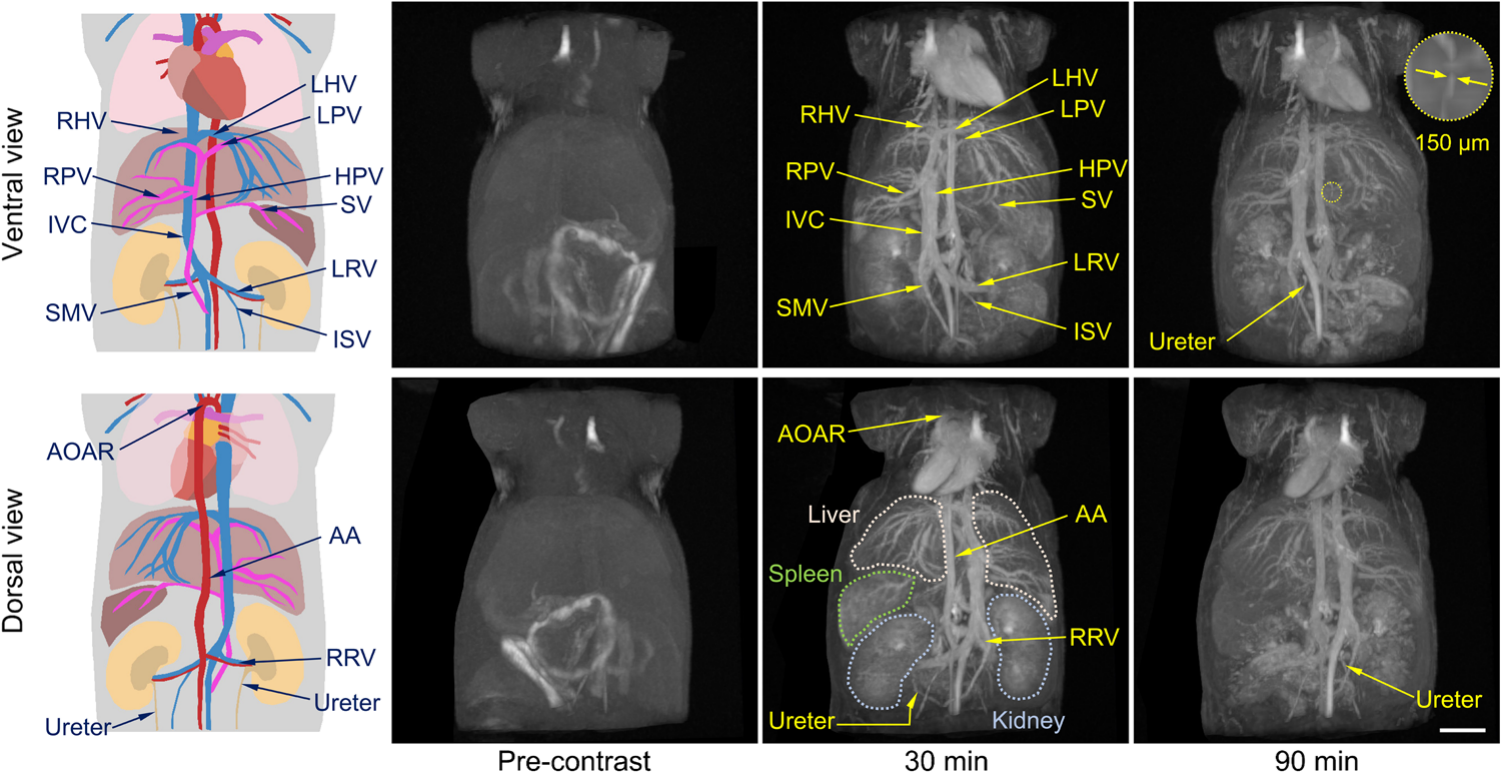
Hepatorenal angiography of rodent animals with PAA-Gd. The ventral view and the dorsal view of schematic drawing of vascular structures (left), and the representative mice angiography obtained pre- and at 30 min, 90 min post-contrast of PAA-Gd through body coil. The embedded scale bar corresponds to 5 mm. Triplicates were performed independently with similar results. Abbreviation: AOAR aortic arch, L/RHV left/right hepatic vein, L/RPV left/right portal vein, HPV hepatic portal vein, SV splenic vein, IVC inferior vena cava, AA abdominal aorta, L/RRV left/right renal vein, SMV superior mesenteric vein, ISV internal spermatic vein.

As aforementioned, hepatic angiography always remains a challenge in MR angiography. In the current design, PAA-Gd can avoid vascular extravasation and immune elimination due to the appropriate hydrodynamic size and the stealth nature of the zwitterionic structure. Therefore, PAA-Gd provides a feasible strategy to dissolve the intractable dilemma between quickly renal clearance and passive parenchyma retention of the contrast agents. The hepatorenal angiography were further acquired with the body coil of mice, 3D MR angiography images were acquired at pre- and 30 min and 90 min post-injection of PAA-Gd, respectively. As shown in Fig. 4 and Supplementary Video 8, the tiny vessels as thin as 150 μm in diameter in liver, mainly the most branches of hepatic vein and hepatic portal vein, can be observed and clearly identified after intravenous injection of PAA-Gd.

More importantly, even at 90 min post-injection, PAA-Gd was still confined to the blood vessel lumen without liver parenchyma extravasation, and the hepatic vessels were still readily distinguished. The relatively low liver accumulation of PAA-Gd could be attributed to the nature of zwitterionic pendant groups on PAA polymer. In addition, according to the MR signals in kidneys at 30 min and 90 min, PAA-Gd entered the medulla from the renal cortex gradually, suggesting that PAA-Gd can be eliminated through urinary system.

### Pharmacokinetics and excretion of PAA-Gd

Pharmacokinetics is one of the key issues related to the clinical translation of contrast agents. Therefore, the pharmacokinetics and excretion of intravenously injected PAA-Gd were investigated.

**Fig. 5:**
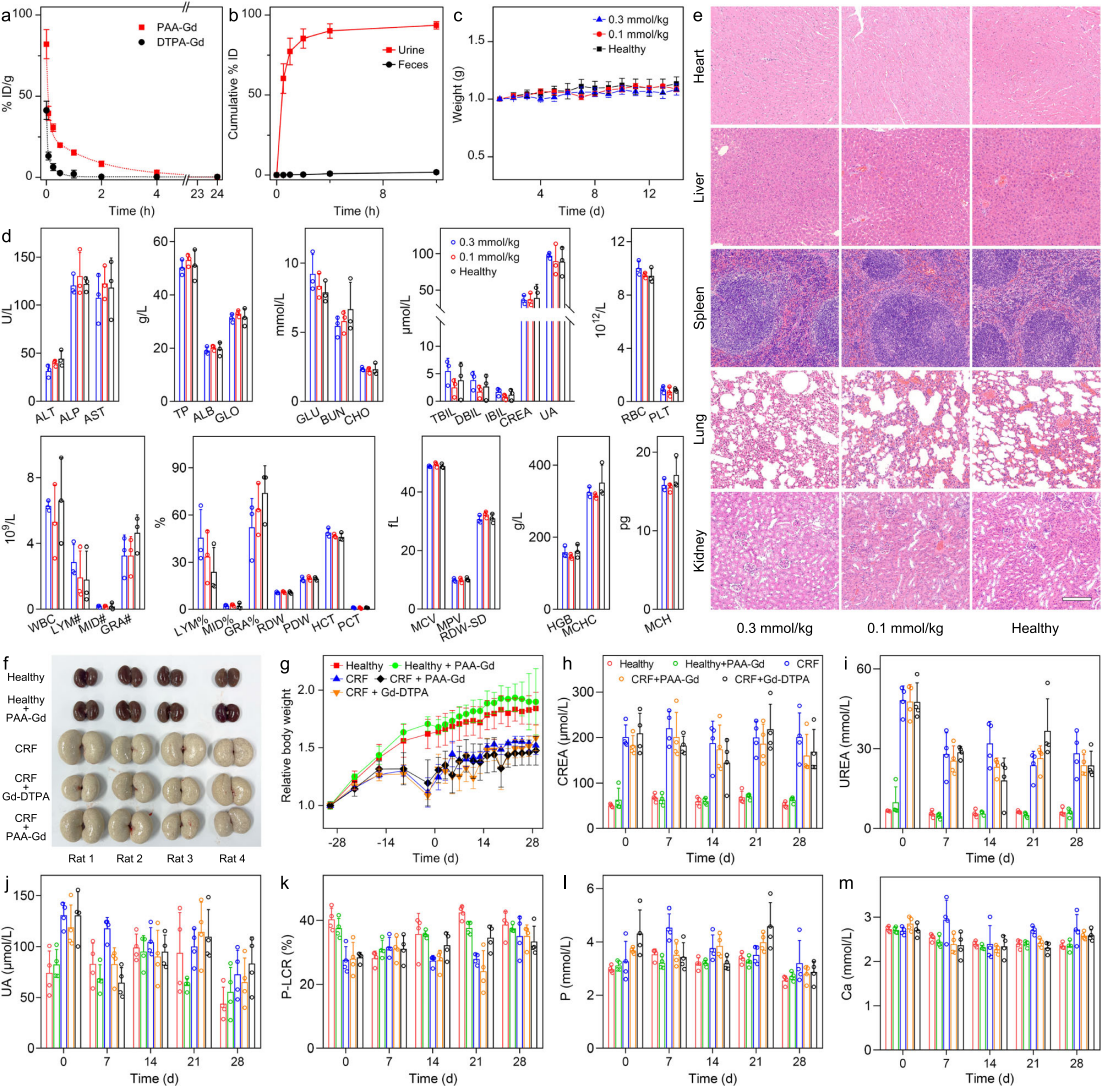
Pharmacokinetics, excretion, and biosafety evaluation of PAA-Gd on rodent animals. **a** Blood clearance profiles of PAA-Gd and Gd-DTPA in BALB/c mice (*n* = 4). **b** Cumulative amounts of PAA-Gd found in feces and urine of mice at different time points post-injection (*n* = 3). **c** Body weight (*n* = 4) and **d** blood test results (*n* = 3) of healthy mice and PAA-Gd-treated mice. **e** H&E staining of the tissue slices of major organs from PAA-Gd-treated mouse. Scale bar: 200 μm. Triplicates were performed independently with similar results. **f** Photographs of the extracted kidneys from healthy rats or CRF rats receiving different contrast agents. **g** Relative body weight, and (**h**–**m**) Blood test results of different groups of rats (*n* = 4). Data were plotted as mean ± standard deviation. Abbreviation: ALT alanine aminotransferase, ALP alkaline phosphatase, AST aspartate aminotransferase, TP total protein, ALB albumin, GLO globulin, GLU glucose, BUN blood urea nitrogen, CHO cholesterol, TBIL total bilirubin, DBIL direct bilirubin, IBIL indirect bilirubin, CREA creatinine, UA uric acid, WBC white blood cell, LYM lymphocytes, MID intermediate cell, GRA granulocyte, RBC red blood cell, PLT platelets, RDW red cell distribution width, PDW platelet distribution width, MCV mean corpuscular volume, MPV mean platelet volume, RDW-SD red blood cell distribution width-standard deviation, HGB hemoglobin, MCHC mean corpuscular hemoglobin concentration, HCT hematocrit, PCT plateletcrit, MCH mean corpuscular hemoglobin, P-LCR platelet large cell ratio.

As an angiography contrast agent, the blood half-life period of PAA-Gd was first characterized by monitoring the Gd concertation in blood by ICP-MS (Thermo, ICAP-Qc) at different time intervals. Through fitting the blood concentrations of Gd^3+^ with a two-compartment model (Fig. 5a), the elimination half-life was calculated to be 73.8 and 11.4 min for the PAA-Gd and Gd-DTPA, respectively. The detailed calculation was provided in the Supplementary Information. The blood half-life period of PAA-Gd is more than six-times longer than that of Gd-DTPA contrast agents.

To further disclose the elimination pathways of PAA-Gd, urine, and feces were continuously collected and quantitatively determined. As shown in Fig. 5b, the time profile of the urinary Gd amount showed that almost all the injected Gd was excreted within the first 4 h post-injection. Over 12 h of urine sampling, the percentage of Gd in collected urine was almost more than 93.5% of the total injected dose, while only 1.8% Gd was detected in feces, thus, it can be concluded that PAA-Gd is excreted through renal filtration. According to our previous studies, the PEGylated NaGdF_4_ nanoparticles with a diameter of either 5.1 nm or 18.5 nm were mainly metabolized through the liver of mice, and most of these nanoparticles will take more than one month to be slowly excreted through the feces. Compared with PEGylated NaGdF_4_ nanoparticles, the fast renal clearance property of PAA-Gd remarkably shortens the retention time of Gd-agent in the body, ensuring their biosafety.

As a comparison, PEGylated NaGdF_4_ nanoparticles were prepared according to our previous studies^38^. Through the transmission electron microscope, size distribution profiles, and DLS provided in Fig. S10, the core diameter and *d*_H_ of PEGylated NaGdF_4_ nanoparticles was 3.6 ± 0.5 nm and 16.1 nm, respectively. Accordingly, the mice injected with PEGylated NaGdF_4_ nanoparticles serves as a positive control, and the healthy mice were set as the negative controls. As a result, although the H&E staining of the tissue slices from all these mice at 24 h post-injection didn’t reflect significant inflammation or injury (Fig. S11), whereas the Chlorophosphonazo III (CPN III) rare earth staining^39^ showed the differences as shown in Fig. S12. The color of all the major organs of both PAA-Gd treated mouse and healthy mouse remained purple, and no green region can be observed, which indicated that there was no Gd deposition in any organ at 24 h post-injection of PAA-Gd. This fact proved the complete clearance of PAA-Gd in mice. However, the heart and liver of NaGdF_4_ nanoparticles-treated mouse were stained green, as well as the kidney slice displayed a blue color in the renal cortex, indicating that the small PEGylated NaGdF_4_ nanoparticles cannot be totally cleared out of the body, and a large part of them still retained in the organs of mouse after 24 h injection.

### Biosafety evaluation of PAA-Gd

In addition to the pharmacokinetics and excretion, the biosafety of PAA-Gd was further evaluated in mice. Firstly, the cytotoxicity of PAA-Gd was investigated on human umbilical vein endothelial cells (HUVECs) through a cell counting kit-8 (CCK-8) assay. As shown in Fig. S13, PAA-Gd presents higher cell viability in comparison with Gd-DTPA, and did not show obvious cytotoxicity even at Gd^3+^ concentration of 10 mM, confirming the good biocompatibility of PAA-Gd.

Apart from the cytotoxicity, the hemocompatibility of PAA-Gd has also been evaluated. As shown in Fig. S14, pure water (positive control) and normal saline (negative control), showed 100% and 0% hemolysis rates, respectively. With respect to the saline solution of PAA-Gd, the hemolysis rates were less than 1.5% even at PAA-Gd concentration of 256 μg/mL, which is more than 1.7-fold higher than the maximum treated blood concentration of PAA-Gd (the circulatory blood volume of a mouse is about 72 mL/kg)^40^. These rates are much lower than the threshold hemolysis rate of 5% set by the International Organization for Standardization and the American Society for Testing and Materials^41^. These results provided a prerequisite indication of safety for the intravenous medication of PAA-Gd.

Furthermore, the in vivo hematological and tissue effects following exposure to PAA-Gd were evaluated on BALB/c mice. Briefly, 12 healthy mice were randomly divided into three groups (*n* = 4). One group of mice was intravenously injected with PAA-Gd contrast agent with the dosage of 0.3 mmol Gd/kg body weight, which is 3 times higher than the common dosage used in clinic. Another group of mice were injected with PAA-Gd with clinical dosage, i.e., 0.1 mmol Gd/kg body weight. The last group of mice without any injection were adopted as controls. At 14 d post-injection, these three groups of mice were sacrificed, and their major organs were extracted and subjected to histological analysis, while the blood samples of 3 mice of each group were collected for blood routine and chemical tests. As shown in Fig. 5c, after injection of PAA-Gd with the dosage of either 0.3 mmol Gd/kg or 0.1 mmol Gd/kg, the mice presented negligible fluctuations in average body weight, which exhibited no significant difference in comparison with the healthy mice, demonstrating no generalized normal tissue toxicity from PAA-Gd administrated.

According to the biochemical and routine blood test (Fig. 5d), there were no significant differences in any serum biochemical parameter at 14 d post-injection compared with the control, and all the parameters remained in the normal range. In addition, no significant hepatic or renal toxicity was observed, as indicated by normal values of liver function markers, including ALT, AST, ALP, TP, ALB, GLO, TBIL, DBIL, and IBIL, and kidney function markers, including BUN, CREA, and UA. Also, neither inflammation-related disease nor acute hemolysis was induced by PAA-Gd, as shown by WBC, LYM#, MID#, GRA#, RBC, PLT, MPV, and PCT. Apart from the blood test, through the H&E staining of the organ slices given in Fig. 5e, no abnormal changes in the pathological histology or cellular structure were observed, indicating that PAA-Gd does not cause any noticeable inflammation or damage in major organs. All above results suggested that the PAA-Gd is a safe contrast agent even at a high dosage level (0.3 mmol/kg) in mice.

In clinical trials, Gd-based contrast agents may lead to adverse side effects on patients with impaired renal function, which has caused widespread concern. In this context, the biosafety of PAA-Gd was further validated on rats with severely impaired renal function. Specifically, adenine-induced chronic renal failure (CRF) rat models were established through gastric gavage of adenine for 30 d. During the model establishment, body weights of the model rats were monitored and compared with the healthy rats, for evaluating the degree of renal impairment. After that, 12 CRF rats were randomly divided into 3 groups. Two groups of rats were intravenously injected with PAA-Gd or Gd-DTPA with a dosage of 0.1 mmol/kg, respectively, and the last group was set as the control group without any injection. Apart from CRF rats, 8 healthy rats were also randomly divided into 2 groups, in which one group was treated with PAA-Gd (0.1 mmol/kg), and the other healthy group was set as the control. After 28 d, the rats were sacrificed, and the kidneys of rats were extracted for histological analysis. As displayed in Fig. 5f, the kidneys from healthy rats and PAA-Gd-injected healthy rats exhibited reddish-brown color with luster, and had a smooth surface without swelling. In contrast, the kidneys from CRF groups are pale and much larger than those extracted from healthy rats, and have dark spots on the surface. To further compare the pathological variation between these groups, the kidney tissues were cut into slices and subjected to H&E staining. As given in Fig. S15, the kidneys from healthy rat and PAA-Gd-injected healthy rat displayed a normal renal tissue structure, uniform tubules lined with a single epithelial layer, and no obvious pathological changes can be observed. In comparison, the kidney tissues from CRF rats exhibited obvious degenerative changes, such as glomerular bulging, glomerular hypertrophy, tubular dilatation, and tubular necrosis. These results suggest that, although the PAA-Gd cannot reverse the renal injury of the rats, there was no significant difference between the kidney tissues from the PAA-injected healthy/CRF rats and non-injected healthy/CRF rats in terms of both renal morphological and pathological characteristics, thus, the PAA-Gd will not lead to the obvious renal damage.

In addition, the body weight variations of the different groups of rats were recorded every 7d during the model establishment period (from day −29 to day 0), and every 2 d after administration of the contrast agents (from day 1 to day 28). As indicated in Fig. 5g, compared to the healthy rats with continuous body weight increase, the weights of adenine-fed rats grew slowly and decreased subsequently. For the healthy rats, the trend of weight increase would not be affected by the PAA-Gd injection. For the CRF model groups, all the rats presented slow weight increases after stopping the adenine gavage. Significantly, the increase trends of both PAA-Gd- and Gd-DTPA-treated CRF rats were same with the CRF rats without injection, suggesting that PAA-Gd and Gd-DTPA would not lead to the deterioration of the renal disease.

Apart from the weight recording, the renal function related-blood indexes of rats from each group, including CREA, UREA, UA, serum phosphorus (P), calcium (Ca), and P-LCR, were determined every week (Fig. 5h–m). As a result, the CREA, UREA, UA, and P-LCR of CRF rats significantly upregulated compared with healthy rats, nevertheless, no significant differences were observed in the upregulation degree of these parameters between the increase degree of PAA-Gd-, Gd-DTPA-injected CKD rats and non-injected CKD rats. In addition, there were no significant variations of serum P and Ca between five different groups of rats.

In clinical trials, the exposure of patients with renal impairment to Gd-based contrast agent may lead to the fibrosis of the connective tissue in the skin and systemic tissues, known as nephrogenic systemic fibrosis (NSF). To dispel this concern, the skin tissues of rats were obtained for histological analysis. According to the H&E staining presented in Fig. S16, no morphological abnormalities were observed in skin tissues, suggesting that the risk of NSF provoked by PAA-Gd is at least not higher than that by Gd-DTPA.

The above results strongly confirmed that the PAA-Gd would not lead to adverse side effect in animals with renal dysfunctions, which highlighted the biosafety of PAA-Gd, and further confirmed the clinical prospect of this contrast agent.

### MR angiography with PAA-Gd in rodent animal models

Benefiting from the long blood-life and higher relaxivity, the MR angiography images with high spatiotemporal resolution can be acquired with the aid of PAA-Gd, to monitor the dynamic variations of vascular anatomic morphology during various treatments such as vascular surgery or thrombolysis therapy in real-time. Herein, PAA-Gd enhanced high-resolution MR angiography was carried out on rodent models of different vascular diseases including carotid thrombosis and plaque, as cases of PAA-Gd-based MRI diagnosis. These cases can further evaluate the applications of PAA-Gd in clinical practice.

### Precise detection of vulnerable atherosclerotic plaque

**Fig. 6:**
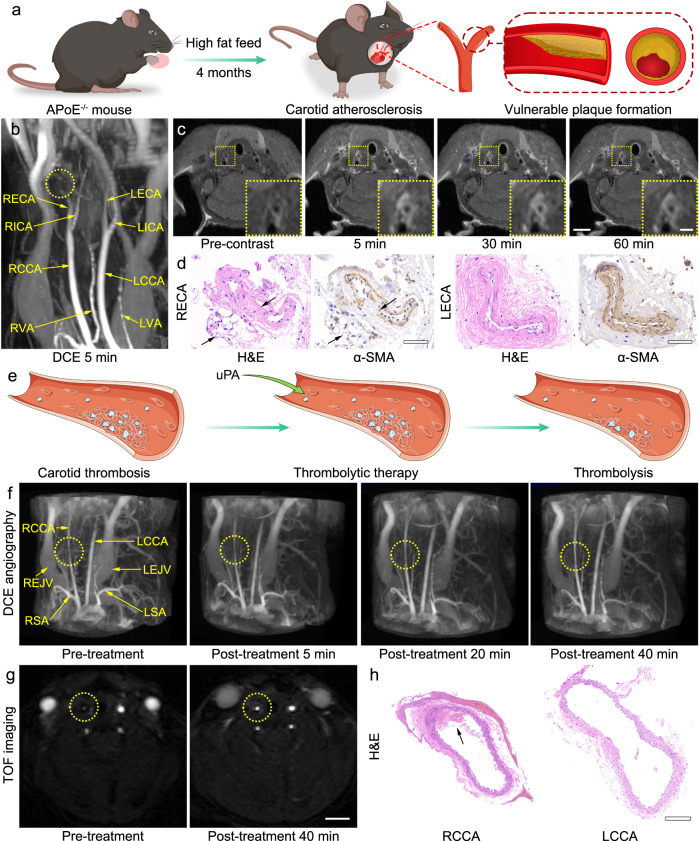
Precise detection of atherosclerotic plaque and arterial thrombosis, and real-time monitoring of thrombolytic therapy. **a** Schematic drawing of the atherosclerosis plaque model establishment. **b** Representative PAA-Gd-enhanced 3D MR angiography at 5 min post-contrast, for showing the vascular stenosis site (yellow dotted circle). **c** Representative *T*_1_-weighted images of plaque section acquired pre- and at different time points post-injection of PAA-Gd. Triplicates were performed independently with similar results. **d** H&E and α-SMA staining of tissue slices from external carotid arteries. Triplicates were performed independently with similar results. **e** Schematic drawing of the thrombosis and thrombolytic therapy. **f** PAA-Gd-enhanced 3D angiography of mouse carotid thrombosis pre-treatment (obtained at 5 min after PAA-Gd injection), and the real-time monitoring of thrombolytic therapy post-treatment of uPA. (The carotid thrombosis were identified by yellow dotted circle). **g** Representative 2D TOF angiography of thrombus section acquired pre- and 40 min post-treatment of uPA. Triplicates were performed independently with similar results. **h** H&E staining of bilateral common carotid arteries. Triplicates were performed independently with similar results. The embedded scale bar of frame **c**, **c** inset, **d**, **g**, and **h**, corresponded to 2 mm, 0.5 mm, 50 μm, 2 mm, and 100 μm. Abbreviation: L/RECA left/right external carotid artery, L/RICA left/right internal carotid artery, L/RCCA left/right common carotid artery, L/RVA left/right vertebral artery, L/REJV left/right external jugular vein, L/RSA left/right subclavian artery.

For atherosclerosis plaque imaging, an animal model with plaque along the carotid artery was established with ApoE^–/–^ mice with high fat and cholesterol diet (Fig. 6a). This animal model was designed to diagnose the vascular stenosis sites by using MRI with PAA-Gd as contrast agent.

Interestingly, apart from 3D diagnosis, PAA-Gd is also expected to diagnose the plaque through 2D *T*_1_-weighted imaging. As known, a patent vessel appears on spin-echo sequences as a signal void because of excited protons leaving the imaging volume before their echo signal is detected. Accordingly, the hallmark of thrombosis is the lack of signal void on enhanced spin-echo sequences and the absence of signal on MRA, known as “flow void effect”. This phenomenon has been regarded as one of the criteria for the clinical imaging diagnosis of thrombosis^42–45^. On this basis, in the PAA-Gd enhanced *T*_1_-weighted imaging, the cross-section of normal carotid artery should show a low signal (black flow void in image). However, after the formation of plaque, this vascular bulge will alter the shear stress patterns of the carotid artery, which will generate vortexes of blood stream and largely reduce the flow void effect^46,47^. Therefore, with the ultra-high longitudinal relaxivity of PAA-Gd, an abnormal hyperintense *T*_1_ signal is expected to appear at the plaque sites to tell us the position of plaque by a positive contrast 2D *T*_1_ weighted imaging at a dark background. Furthermore, in 3D MR angiography, the plaque could be identified at the same site as a negative contrast, which allows a dual identification strategy to improve the diagnostic accuracy.

To confirm this hypothesis, in vivo imaging studies were carried out in the animal atherosclerosis model before and after intravenous injection of the PAA-Gd. The 3D MR angiography was firstly conducted, as shown in Fig. 6b, after injection of PAA-Gd, a stenosis of blood signal can be observed at the right external carotid artery (yellow dotted circle). In addition, the *T*_1_-weighted MR images shown in Fig. 6c revealed that in comparison with the flow void of the left carotid arteries, the vascular wall and its periphery of the right external carotid artery was quickly brightened after post-injection, and this enhanced contrast could last for over 60 min. Based on these dual-modalities plaque imaging, the abnormal sites at right external carotid artery can be surely identified as the suspected plaque. Note that each scan takes only 5–6 min, it is possible to improve the image quality by averaging multiple-time scans resulting in high diagnostic accuracy.

For verifying these plaque imaging results, the histological studies were conducted right after the MR imaging studies (Fig. 6d). Typical features of atherosclerotic plaque morphologies including cholesterol crystals and accumulation of foam cells (indicated by the black arrows) were observed in hematoxylin-eosin (H&E) staining of carotid tissue isolated from the corresponding region of the right external carotid artery of the imaging mouse. Immunohistochemical analysis of alpha-smooth muscle actin (α-SMA) revealed that the vascular smooth muscle cells (VSMCs) migrate from the medial layer into the intima, which further confirmed the formation of the plaque. In addition, the expression of α-SMA of VSMCs in the plaque region was down-regulated in comparison with that of contralateral healthy vessels, implying that this plaque may be vulnerable^48^. These results supported the PAA-Gd enhanced MR imaging of plaques detection.

### In vivo 3D MR angiography of arterial thrombosis and the real-time monitoring of thrombolytic therapy

Thrombosis is another type of cardiovascular/cerebrovascular-associated diseases, which is associated with vascular injuries of any causes. When the vessel wall is breached or the endothelium is disrupted, collagen and tissue factor become exposed to the flowing blood, which subsequently trigger the activation of platelets and initiate the generation of thrombin, thereby initiating formation of a thrombus. At the sites of thrombus, acute blood vessel obstruction can lead to severe tissue damage or even organ failure, ultimately becoming life-threating. Therefore, specific and effective removal of thrombus to recanalize blood vessels and restore blood supply is the key to thrombotic diseases treatment.

At present, intravenous injection of plasminogen activators, such as urokinase (uPA), streptokinase (SK), and recombinant tissue plasminogen activator (rt-PA), is a commonly used clinical thrombolysis method. These plasminogen activators act on the fibrinolytic system to trigger the lysis of water-insoluble fibrin to play a thrombolytic role (Fig. 6e). Notably, due to the short half-life of plasminogen activator uPA treatment, it is needed to continuously inject uPA in the clinic to ensure a long-term blood concentration, but it increases the hemorrhage risk^49,50^. Although this thrombolytic therapy has been adopted extensively, the systemic administrated fibrinolytic drugs may disrupt the normal balance between fibrinolytic and coagulation system, resulting in serious bleeding complications like intracranial hemorrhage.

In this context, the dosage of fibrinolytic drugs should be strictly controlled. In particular, the lysis of thrombus during the intravenous infusion of thrombolytic drugs is expected to be monitored in real-time and stopped in time to prevent hemorrhagic complications. In order to monitor the vascular structure during the thrombolysis therapy, MR angiography based on PAA-Gd has also been performed with a FeCl_3_-induced carotid arterial thrombosis mouse model.

As shown in Fig. S17, after surgery with FeCl_3_, the formation of right-sided carotid arterial thrombosis was proved by TOF MR angiography, which showed that the surgical site of blood vessel was stenotic with very few hemodynamic signals in comparison with the contralateral normal carotid artery.

After the intravenous injection of PAA-Gd through the tail vein, the 3D MR angiography of the mouse neck was acquired. As shown in Fig. 6f and Supplementary Video 9, the arteries and veins of mice including carotid artery, jugular vein, aortic arch, and even vertebral artery were clearly depicted after PAA-Gd injection. More importantly, significant stenosis could be clearly observed in the middle segment of the common carotid artery (indicated by the yellow dotted circle), suggesting that 3D MR angiography based on PAA-Gd can realize the precise positioning of the thrombus.

As known, TOF provides morphologic information about the vessels, relying on blood flow as the physical basis for generating contrast between stationary tissues and moving spins. Therefore, the TOF technique provides a maximal signal only when blood flow is perpendicular to the imaging plane. Post-stenotic segments of blood vessels exhibit disordered or turbulent flow that results in dephasing, in which the different velocity components with different phases that would cancel each other out and result in no signal. This decrease in signal intensity manifests as an exaggeration of either the degree or the length of stenosis or both. It has been demonstrated that TOF MRA is degraded by turbulence-induced signal loss in regions of stenosis, often leading to stenosis over-grading, and stenosis can even be falsely indicated as occlusion^51–55^. By comparison, the intravascular signal is mainly dependent on *T*_1_ shortening of the blood rather than flow-related enhancement in PAA-Gd enhanced MRA, therefore, the accurate morphological of vascular stenosis can be better delineated by PAA-Gd enhanced MRA.

To further evaluate the feasibility of PAA-Gd enhanced MR angiography in guiding the thrombolysis therapy, uPA was adopted to dissolve the thrombus through intravenous injection subsequently. As shown in Fig. 6f, the blood flow of the right-sided carotid arterial gradually recovered within 40 min post-injection of uPA, although it did not completely recover to the same level as the left carotid. This result was further confirmed by the 2D TOF MR angiography, in which the hemodynamic signals of the right-sided carotid artery significantly recovered, but this signal was still lower than the left one (Fig. 6g)

For further verifying the imaging results, the histological studies were conducted right after the MR imaging studies. The surgical segment of right-sided carotid tissue, and the same segment of left-sided carotid tissue were extracted from the imaging mice. According to the H&E staining (Fig. 6h), the injury of vascular endothelial cells and the intraluminal thrombosis can be clearly observed, strongly supporting the above thrombus imaging results. Therefore, the time window of MR angiography with PAA-Gd matches the duration of this thrombolytic therapy so perfectly, that it can monitor the whole process of treatment and warn thrombolysis instantly.

### MR angiography with PAA-Gd in large mammal animal model

**Fig. 7:**
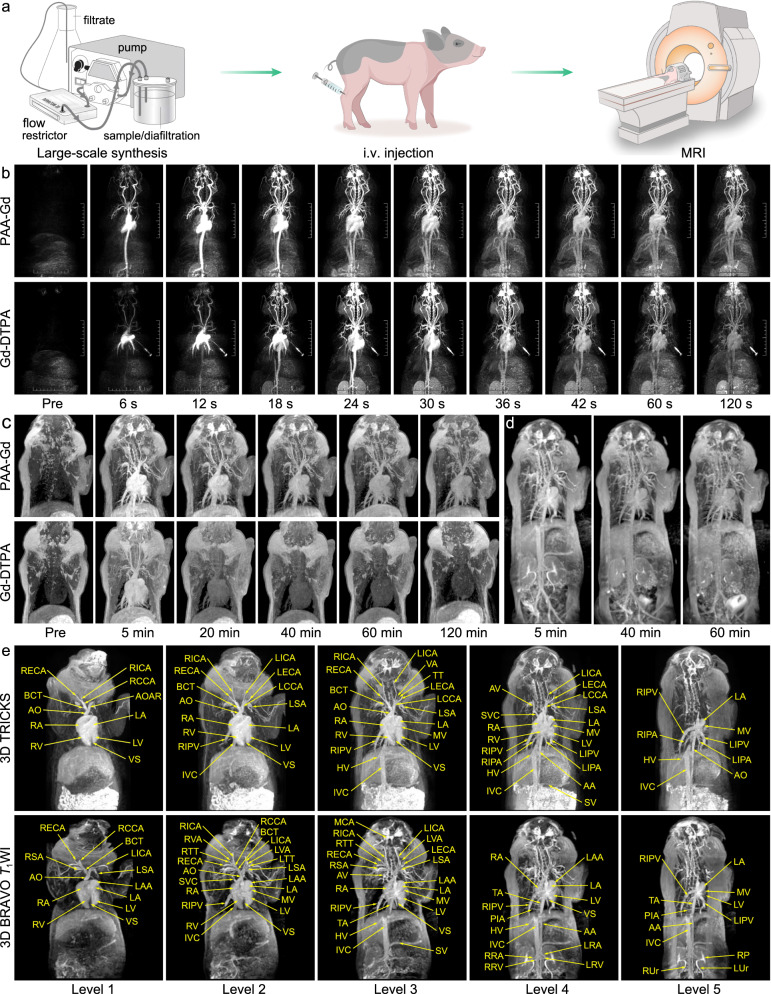
MR angiography of swine with PAA-Gd and vascular identification. **a** Schematic illustration of large-scale synthesis of PAA-Gd and MR angiography procedure. **b** Real-time 3D TRICKS MR angiography obtained pre- and post-injection of PAA-Gd with 0.03 mmol/kg (upper) and Gd-DTPA with 0.1 mmol/kg (lower). **c** Delayed 3D TRICKS MR angiography obtained pre- and post-injection of PAA-Gd with 0.03 mmol/kg (upper) and Gd-DTPA with 0.1 mmol/kg (lower). **d** Delayed 3D BRAVO *T*_1_-weighted MRI obtained at different time points post-injection of PAA-Gd with 0.03 mmol/kg. **e** Different coronal levels of 3D TRICKS angiography (upper) and 3D BRAVO images (lower) for identifying the vascular structures. Abbreviations: MCA middle cerebral artery; AO aorta, AOAR aortic arch, BCT brachiocephalic trunk, L/RECA left/right external carotid artery, L/RICA left/right internal carotid artery, LCCA left common carotid artery, L/RVA left/right vertebral artery, L/RSA, left/right subclavian artery, VA vertebral artery, L/RVA left/right vertebral artery, TT thyrocervical trunk, L/RTT left/right thyrocervical trunk, SVC superior vena cava, L/RIPA left/right inferior pulmonary artery, L/RIPV left/right inferior pulmonary vein, IVC inferior vena cava, HV hepatic vein, TA thoracic aorta, AA abdominal aorta, PIA posterior intercostal artery, AV azygos vein, SMV superior mesenteric vein, SV splenic vein, L/RRA left/right renal artery, L/RRV left/right renal vein, L/RA left/right atrium, L/RV left/right ventricle, VS ventricular septum, MV mitral valve, LAA left atrial appendage, RP renal pelvis, L/RUr left/right ureter.

In order to further evaluate the clinical translation prospect of PAA-Gd, the 3D MR angiography in larger mammal animal, bama miniature swine, has also been conducted on a 3.0 T clinical human MRI instrument (Fig. 7a). Two clinical contrast-enhanced MRI sequences, three-dimensional time-resolved imaging of contrast kinetics (3D-TRICKS) and three-dimensional BRAin VOlume *T*_1_ imaging (3D BRAVO), were adopt for showing the anatomy of swine vessels and tissues. Specifically, 3D-TRIKES is a clinical sequence for fast hemodynamic acquisitions, which is expected to obtain DCE-MR images at both high-spatial and high temporal resolution in swine^56^; 3D BRAVO is a clinical fast *T*_1_-weighted 3D gradient-echo (GRE) sequences, which has been commonly used for high-spatial-resolution human cerebrovascular and brain diseases imaging^57^.

Firstly, the same dose (0.1 mmol per kg body weight) of PAA-Gd and Gd-DTPA was intravenously injected into the swine after anesthesia through small saphenous vein of hind leg, respectively. The 3D TRICKS MR images acquired pre- and post-injection of PAA-Gd and Gd-DTPA were given in Fig. S18. Most surprisingly, the enhancement of PAA-Gd in MR angiography signals was much higher than Gd-DTPA. This result encouraged us to challenge the angiography of swine with one-third dosage of PAA-Gd (0.03 mmol per kg body weight).

In clinical, early phase MRA is of great significance, because it can reflect the arterial anatomy more clearly to diagnose the different arterial diseases such as steno-occlusive lesions, aneurysms, etc. In this context, the temporal evolution of enhanced TRICKS of swine during the first 120 s after injection were conducted. As shown in Fig. 7b, after the intravenously injection, PAA-Gd (0.03 mmol/kg) returned to the heart with the blood flow, and was pumped through the aorta out to the body to brighten the different levels of arteries, including arteria coronaria, carotid arteries and the branches, subclavian arteries, abdominal aorta, and renal arteries, etc. within the first 12 s after injection (arterial phase). After that, PAA-Gd enters all levels of veins to further depict the venous system in the next phases (venous phase). By contrast, Gd-DTPA (0.1 mmol/kg) could also display the arteries during the first 12 s (arterial phase), however, this low-molecular-weight contrast agent rapidly accumulated in the kidney after the first-pass phase, which reduced the blood concentration of agents, leading to an insufficient resolution in venous angiography. Notably, the differences of PAA-Gd and Gd-DTPA in enhancement of pulmonary vessels and intracranial to abdominal aorta in the first 18 s, may due to the different dilution behaviors of these two contrast agents in the bloodstream. According to the Stokes-Einstein equation^58^, due to the large hydrodynamic diameter, the PAA-Gd have smaller diffusion coefficient than Gd-DTPA (PAA-Gd 5.4 × 10^−11^ m^2^/s; Gd-DTPA 5.7 × 10^−10^ m^2^/s), so PAA-Gd can maintain more concentrated fluid for a relative longer time in blood after injection. Therefore, after passing the pulmonary circulation, the blood containing undispersed PAA-Gd can be pumped out to cerebral vessels and abdominal aorta. In contrast, when small-molecular-weight Gd-DTPA was pumped into the lungs from the heart, the Gd-DTPA would disperse quickly, thus more pulmonary vessels could be displayed in the first few seconds after injection.

The temporal evolution of the vascular signal intensities in TRICKS during the early phases (~120 s) were quantitatively analyzed. As shown in Fig. S19, only 1/3 dosage of PAA-Gd can produce a strong arterial signal after injection, which is comparable with Gd-DTPA with a full dosage. However, owing to the quickly elimination of Gd-DTPA, both the arterial and venous signals enhanced by Gd-DTPA declined very quickly. More importantly, compared with Gd-DTPA, the time difference between arterial signal peak and venous signal peak after PAA-Gd injection is much longer. This longer delay of venous in the maximum signal caused by PAA-Gd provided long enough time window to separate the arteries, allowing the better distinguishing between arteries and veins. Overall, the short acquisition time of single imaging phase of TRICKS and higher *r*_1_ of PAA-Gd make it possible to obtain purely arterial phase images. The long angiography window of PAA-Gd can also ensure the venous depiction in the next delayed phases. This result suggested that PAA-Gd can provide more clinically helpful information of arterial and venous diseases than Gd-DTPA.

Following the angiography of first 120 s, the delayed phase of MRA were also obtained, as given in Fig. 7c. At 5 min post-injection of PAA-Gd with the dose of 0.03 mmol Gd /kg, a high-resolution 3D angiography of the upper body in swine could be obtained, and the vascular structure of swine was clearly identified, indicating the high-spatial resolution of the vasculature. In contrast, Gd-DTPA with the dose of 0.1 mmol Gd/kg can also depicted the distribution of blood vessels at 5 min post-injection, but the vascular contrast was much lower than that of PAA-Gd due to the extravasation of Gd-DTPA in the perivascular tissues. The average *T*_1_ signal-to-background ratios between five different blood vessels and their surrounding tissues were calculated. As shown in Fig. S20, the PAA-Gd enhanced MR angiography has much higher vascular signal-to-background ratio than Gd-DTPA-enhanced one (*p* < 0.01) at 5 min post-injection, which highlights the angiography performance in the delayed phase of the PAA-Gd. More importantly, the blood distribution of swine treated with PAA-Gd can still be clearly distinguished at 120 min post-injection, PAA-Gd was still restricted in vessels without extravasation, even if the MR signal of blood vessels faded gradually. In contrast, the vascular signal enhanced by Gd-DTPA nearly vanished within only 20 min post-injection. The quantitatively analysis of MR signal temporal evolution of blood vessels and the surrounding tissues were shown in Fig. S21. Accordingly, only 1/3 dosage of PAA-Gd can give a much higher MRI signal than DTPA after injection, and maintained a substantially stronger MRI signal in its steady state. This result agrees well with the previous reported MR angiography results in Fig. 2a. The long-term circulation of PAA-Gd is able to provide a enough time window for vascular imaging.

For further evaluating the elimination of PAA-Gd in the swine, the temporal evolution of 3D BRAVO of the whole body before and after intravenous injection of PAA-Gd was acquired. As shown in Fig. 7d and Fig. S22, the blood vessels of swine injected with PAA-Gd can also be clearly observed through this MR sequence. More importantly, over the course of 2 h, the contrast of bladder is significantly increased (the temporal evolution of the relative signal intensity of bladder is provided in Fig. S23), which confirms that PAA-Gd was renally filtered from blood vessels and would be excreted from the body in urine eventually. Accordingly, the excretion pathway of PAA-Gd in swine is consistent with that in mice, ensuring the biosafety of PAA-Gd in large animals. By contrast, in the delayed Gd-DTPA-enhanced BRAVO imaging shown in Fig. S24, the anatomical structure of vessels is ambiguous and the microvessels cannot be distinguished after 5 min post-injection.

Based on the anatomic atlases, the swine cardiovascular structures in the probe-enhanced MR angiography were carefully identified with their name abbreviations. According to Supplementary videos 10 and 11, the spatial evolution of blood vessels of swine from the ventral to dorsal levels can obviously display every single vessel in the swine body. For ease of identification in the plane figures, the heart and peripheral blood vessels in 3D TRICKS and 3D BRAVO images were divided into 5 coronal levels with same thickness. The sophisticated blood vessels of each level were identified. As shown in Fig. 7d, the morphology of blood circulation systems, including the structures of atrium, ventricles, and valves; the main arteries originated from the heart; the main veins returned to the heart; and the different levels of head, neck, and abdominal blood vessels, can be clearly identified. As the controls, clinically used none enhanced angiography sequence, including Oblique Axis TOF MRA and Sagittal 3D Phase-Contrast (PC) MRA that have been regarded as the most popular non-contrast-enhanced MRA techniques in clinical practice, were also adopted to show the vascular structure of swine, as shown in Figs. S25 and S26. However, due to the flow-dependent properties of these two sequences, only the large blood vessels with fast blood flow velocity can be observed. Besides, the complex vascular systems were hard to distinguish, especially for the microvessels that are not perpendicular to the scanning orientation in TOF angiography and the cardiac blood flow with frequent velocity and magnitude changes in PC angiography. In addition, the long image acquisition time of TOF and PC caused respiratory motion artifacts, which further interfered with cardiovascular angiography and would largely hamper the accurate depiction of diseased vascular anatomy. Overall, in comparison with TOF and PC angiography, the advantages of PAA-Gd enhanced MRA include shorter acquisition times, improved anatomical coverage, and decreased susceptibility to artifacts caused by blood flow and pulsatility. Additionally, TOF and PC can only display the main vessels with enough blood flow velocities perpendicular to the imaging plane.

Most of these limitations can be well addressed with PAA-Gd-enhanced MR angiography. Instead of relying on blood motion to create intravascular signal, PAA-Gd will enhance the *T*_1_ relaxation of blood for a long period after intravenous injection. Therefore, the blood can be directly imaged irrespective of flow velocity in a long time window. Overall, PAA-Gd enhanced 3D angiography undoubtedly makes a great leap in vascular visualization, which possesses great clinical application prospects.

The above results indicated that PAA-Gd exhibits outstanding MR angiography performance in large animals, which is even better than that in the rodent model. It thus can be reasonably speculated that PAA-Gd is able to competent for the diagnosis of multiple vascular diseases in large animals. In addition, it should be mentioned that the angiography performance achieved by 1/3 clinical dose of PAA-Gd is still much better than the full dose of Gd-DTPA. This property will largely dispel the concern about the side effects of Gd-based contrast agents due to potential Gd^3+^ deposition. Overall, PAA-Gd has excellent prospects for clinical translation in the field of vascular disease diagnosis.

### Biosafety evaluation of PAA-Gd on large animals

**Fig. 8:**
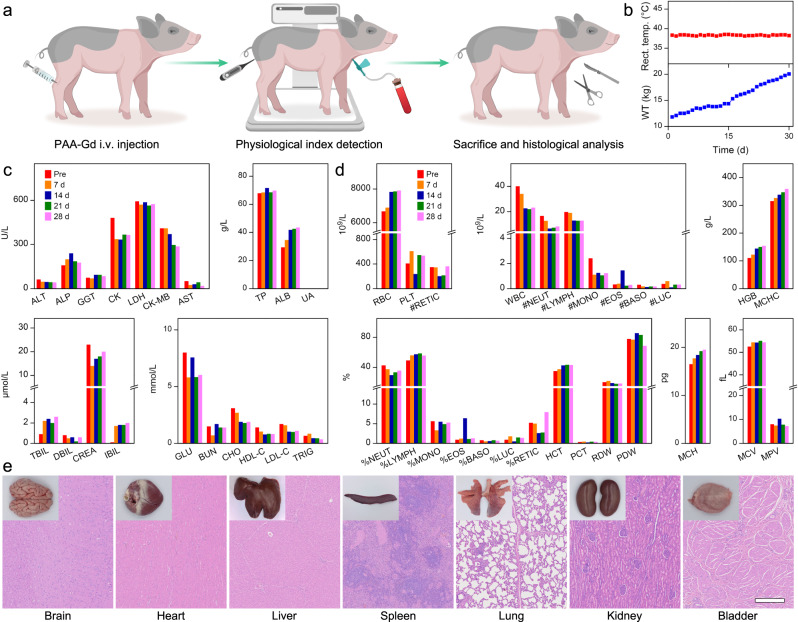
Biosafety evaluation of PAA-Gd on swine. **a** Schematic illustration of PAA-Gd injection, physiological index detection, and histological analysis process. **b** Fluctuations in the rectal temperature and the increase of body weight of sexually mature swine after PAA-Gd administration. **c** Blood biochemical test results of sexually mature swine treated with the PAA-Gd contrast agents. **d** Routine blood test results of sexually mature swine treated with the PAA-Gd contrast agents. **e** H&E staining of tissue slices from major organs of sexually mature swine treated with the PAA-Gd at 30 d post-injection. Triplicates were performed independently with similar results. The embedded scale bar corresponded to 500 μm. Data were plotted as mean ± standard deviation. Abbreviation: ALT alanine aminotransferase, ALP alkaline phosphatase, GGT gamma-glutamyl transpeptidase, CK creatine kinase, LDH lactate dehydrogenase, CK-MB creatine kinase isoenzymes, AST aspartate aminotransferase, TP total protein, ALB albumin, UA uric acid, TBIL total bilirubin, DBIL direct bilirubin, CREA creatinine, IBIL indirect bilirubin, GLU glucose, BUN blood urea nitrogen, CHO cholesterol, HDL-C high density lipoprotein cholesterol, LDL-C low density lipoprotein cholesterol, TRIG triglyceride, RBC red blood cell, PLT platelets, RETIC reticulocyte, WBC white blood cell, NEUT neutrophil, MONO monocyte, EOS eosinophils, BASO basophil, LUC large unstained cell, HGB hemoglobin, MCHC mean corpuscular hemoglobin concentration, LYMPH lymphocytes, HCT hematocrit, PCT plateletcrit, RDW red cell distribution width, PDW platelet distribution width, MCH mean corpuscular hemoglobin, MCV mean corpuscular volume, MPV mean platelet volume.

The biosafety of PAA-Gd in swine was also evaluated. As shown in Fig. 8a, a sexually mature swine was injected with PAA-Gd with the dosage of 0.1 mmol/kg with respect to Gd. The behavior of the young swine after imaging experiments and the PAA-Gd injected sexually mature swine were monitored and recorded daily for more than one month. The swine exhibited no abnormalities in eating, drinking, urination, or neurological status during the experimental period. As shown in Figs. S27 and 8b, after injection of PAA-Gd, the body weight of young swine and mature swine continued to increase from 4.60 kg to 10.05 kg and 11.80 kg to 20.05 kg within one month, respectively, and the rectal temperature of these two swine were stable without abnormal rise or fall. In addition, the whole-blood and serum analyses were conducted on the swine before and at several time points after the injection of the PAA-Gd to evaluate the potential side effects of PAA-Gd on swine (Figs. S28, 29 and Fig. 8c, d). Compared with the values of these indexes pre-injection, there was no sharp increase or decrease in any hematological or serum biochemical parameter during one month after injection, and all the parameters remained in the normal range after one month. After one month, the two swine were sacrificed and their organs, including heart, liver, spleen, lung, kidney, brain, and bladder, were extracted and fixed with formalin. Paraffin sections of the tissues were stained with H&E to evaluate the possible tissue injury induced by injection of the PAA-Gd. As shown in Fig. S30 and Fig. 8e, no abnormal changes in the pathological histology or cellular structure were observed, and none of the organs showed hemorrhage, inflammation, or necrosis, indicating no obvious risk of the PAA-Gd to swine following intravenous injection. In addition, CPN III rare earth staining of the organ slices from mature swine receiving 0.1 mmol/kg PAA-Gd was also carried out, as shown in Fig. S31, no Gd^3+^ signal can be detected in the organs at 30 d post-injection, which indicated that PAA-Gd have been completely eliminated from the body. The above evaluations strongly confirm the biosafety of PAA-Gd on large animals, which provides a solid base for its clinical translation.

## Discussion

On the basis of the above in vitro and in vivo studies, the clinical translational prospect of PAA-Gd was further evaluated and discussed. Firstly, for the main components of PAA-Gd, PAA has been regarded as a non-toxic and non-irritant excipient in the development of biocompatible injectable thermoplastic oligomers^59,60^. DTPA is the approved pharmaceutical starting material for the manufacture of clinically used Gd-DTPA contrast agent injections. Therefore, the resultant PAA-Gd possesses good potential to be employed in the clinical practice of MRA in the future.

Secondly, the simple synthetic procedure of PAA-Gd makes it possible to be prepared with large scale for large animals. The digital photograph of practical equipment of the large-scaled PAA-Gd synthesis system has been shown in the left panel of Fig. S32. Each step of reaction shown in Scheme 1 can be finished in large-volume reactors, and the resultant mixtures of each step can be transferred into the diafiltration reservoir to be purified. After flowing through the flow restrictor, the unconnected small-molecule reactants can be filtered into a conical flask, so that the polymers can be concentrated and purified with the increase of the number of cycles. Through using this system, PAA-Gd can be prepared and purified on a large scale, thereby achieving a gram-level mass production. As shown in the right panel Fig. S32, after the removal of endotoxin, the resultant PAA-Gd can be packaged standardly for further clinical use. During the production, no special device is required for the preparation of PAA-Gd compared with other Gd-based injections. Therefore, the preparation process for PAA-Gd has a good chance of being approved for pharmaceutical applications.

Thirdly, the biosafety of PAA-Gd were carefully evaluated, as shown in Figs. 5 and 8. In rodent animals, no adverse side effects were observed in mice after the administration of PAA-Gd with clinical dosage (0.1 mmol/kg) and 3-fold of clinical dosage (0.3 mmol/kg). Moreover, the CRF rats didn’t show any obvious aggravation in 1 month after receiving the PAA-Gd. Additionally, in the large animal, PAA-Gd would not lead to any adverse side effect in the swine either with imaging dosage (0.03 mmol/kg) or clinical dosage (0.1 mmol/kg). All these results highlighted the biosafety of PAA-Gd. Overall, the high biosafety and convenient large-scale synthetic route endow the PAA-Gd with great clinical translation prospects.

In the aspect of MRI, both the TRICKS MRA sequence and the BRAVO *T*_1_ sequence were adopted for visualizing the anatomical structure of vascular systems of swine. In comparison with the TRICKS MRA sequence with a scan time for only several seconds, the BRAVO *T*_1_ sequence possess higher imaging resolution, though the scan time of BRAVO sequences is much longer (~3–4 min).

The PAA-Gd and Gd-DTPA enhanced TRICKS imaging at the early phases, and the TRICKS/BRAVO imaging at the delayed phases were conducted, respectively. As shown in Fig. 7, Figs. S22 and S24, PAA-Gd can clearly depict the vascular structure both in the early phase through TRICKS sequences, and the delayed phases of TRICKS and BRAVO sequences. Additionally, more microvessels can be identified in the PAA-Gd enhanced BRAVO images due to the higher resolution of BRAVO sequence (Fig. 7e). By contrast, Gd-DTPA with temporal resolution of 15–120 seconds can only depict the vascular structure in the early phase of TRICKS, but hard distinguished the vessels either in BRAVO or TRICKS in delayed phases after 5 min of injection.

Therefore, the clinical MRA with conventional low-molecular-weight Gd contrast agents is limited by the need to acquire images relatively quickly during the first pass of contrast agents through the vessels of interest. In the current study, the bloodstream retention of PAA-Gd allows higher resolution angiography of long-scan-time sequences, such as BRAVO, which may be of great significance in the accurate diagnosis of microvascular diseases, especially for the micro-venous diseases.

In addition to Gd-DTPA contrast agent, Ferumoxytol is an agent based on ultrasmall superparamagnetic iron oxide (USPIO) initially approved by the Food and Drug Administration (FDA) for the iron replacement therapy of patients with anemia due to chronic renal failure. In recent years, Ferumoxytol has been investigated extensively as an intravenous contrast agent in MRA. Similar with PAA-Gd, Ferumoxytol has a long intravascular half-life, making it a potentially useful agent for vascular and perfusion-weighted MRI. However, the biodistribution and clearance of USPIO and PAA-Gd is much different. USPIO will be taken up by the reticuloendothelial system of the spleen, bone marrow, and especially liver (up to 70% of injected dose) after eliminated from the blood, whereas PAA-Gd eliminates by the urinary system after an appropriate residence within the blood pool. In the previous clinical studies, Ferumoxytol may retained in the liver for >3 months, during which the diagnostic ability of MRI would be interfered^61,62^. In this context, the organ retention of Ferumoxytol will make it difficult to continuously monitor the disease progress or treatment effect through enhanced MRI in a relatively short time period. In fact, it has been reported that after injecting a standard dose of Ferumoxytol, the follow-up MRI diagnosis of patient was seriously affected due to the residual hyperintense *T*_1_ signal^63^. In addition, owing to the *T*_2_ effect of superparamagnetic iron oxide, although the Ferumoxytol exhibits high *T*_1_ signal at low concentration, this *T*_1_ signal will be largely weakened at high concentration. In this context, susceptibility-related signal loss can be observed in the abdominal aorta and even the portal vein with high Ferumoxytol concentration during the first pass^64^. By contrast, paramagnetic Gd-chelates contrast with lower *r*_2_ can still possess strong *T*_1_ signal even at relatively high concentration. In fact, both PAA-Gd and Ferumoxytol contrast agents are useful MRA contrast agents, which should be rationally selected for use due to their different advantages and limitations.

In summary, a strategy of hypersensitive MR angiography with high resolution was successfully developed on the basis of a zwitterionic polymeric contrast agent PAA-Gd. With the ideal *d*_H_ and zwitterionic structure, PAA-Gd possess appropriate blood half-life and can be confined in the vascular lumen without extravasation, as well as low immunogenicity to avoid being captured by RES system. The prolonged *τ*_R_ of Gd^3+^ in PAA-Gd endow it with ultra-high *r*_1_, thereby making a qualitative leap to MR angiography. On the rodent animal models, the sophisticated and tiny blood vessels can be clearly visualized through PAA-Gd-enhanced MR angiography on an animal MRI scanner. Based on this performance, the vascular variation in ischemic stroke, atherosclerotic plaque, and thrombosis/thrombolytic process can be depicted with high resolution for achieving precisely diagnosis and prognosis evaluation. On swine model, a great range of clinically important vessels can be clearly identified through MR angiography enhanced with PAA-Gd at as low as 1/3 of clinical dosage on a clinical MRI scanner. It thus can be reasonably believed that PAA-Gd is able to competent for the diagnosis of multiple vascular diseases in large animals, just like in rodent models. More importantly, PAA-Gd can be quickly eliminated through the urinary system without any undesired organ retentions in both rodent animals and swine, highlighting their biosafety features.

With satisfactory angiography performance and biosafety, PAA-Gd could potentially serve as the next-generation MR angiography contrast agent to assist comprehensive morphological investigations of a variety of complicated blood vessels and vascular diseases, not limited to the vessels displayed in the current study.

## Methods

### Materials

Sodium polyacrylate (PAANa, Mw: 5400) was purchased from Sigma-Aldrich. Gadolinium chloride hexahydrate (GdCl_3_·6H_2_O, 99.9%), diethylenetriamine (DET), DTPA, and 4-(4,6-dimethoxy-1,3,5-triazin-2-yl)−4-methyl morpholinium chloride (DMTMM) were purchased from Aladdin Co. Ltd. Sodium hydroxide (NaOH, 96%), Sodium bicarbonate (NaHCO_3_) and sodium citrate was obtained from Beijing Chemical Reagents Co. Ltd. Human umbilical vein endothelial cells (HUVECs) were purchased from American Type Culture Collection (ATCC).

### Cell culture

Human umbilical vein endothelial cells (HUVECs) were cultured in Endothelial Cell Medium (ECM) with 5% fetal bovine serum (FBS), 1% endothelial cell growth factor (ECGF), and 1% penicillin/streptomycin Solution at 37 °C under 5% CO_2_ atmosphere. The cell line was tested negative for mycoplasma contamination by the mycoplasma detection kit (Yeasen Cat. No. 40611).

### Cell viability assays

Cell viability was determined by Cell Counting Kit-8 (CCK-8) assay. Specifically, HUVECs were seeded into a 96-well cell culture plate with a density of 5 × 10^3^ cells/well under 100% humidity, and then cultured at 37 °C in an atmosphere containing 5% CO_2_ for 12 h. Then, the PAA-Gd or Gd-DTPA with different concentrations were introduced into the wells. The following incubation was lasted for 24 h at 37 °C under 5% CO_2_. After the supernatant containing the excrescent Gd-based agents was decanted, the cells were incubated for another 48 h. After that, 10 μL of CCK-8 was added to each well, and incubated for 4 h at 37 °C. Thereafter, the optical density of each well at 450 nm was recorded on a microplate reader (Thermo, Varioskan Flash).

### Hemolysis test

Hemolysis test was conducted according to the conventional method. Briefly, a 2 mL blood sample was mixed with 6 mL of normal saline (NS) and centrifuged at 1500 rpm for 15 min. The supernatant was drawn off and five subsequent washes were carried out before diluting the sample to a final volume of 1 mL in NS. The red blood cells were then diluted 1: 4 into NS solutions (negative control); water (positive control); PAA-Gd solutions (in NS) at varying concentrations (tested samples).

Thereafter, the samples were left at room temperature in the dark for 4 h at 37 °C and then centrifuged at 3000 rpm for 5 min. After centrifuging, the supernatant was transferred to a 96-well plate and the absorbance at 541 nm was recorded.

The hemolysis rate was calculated by the following equation:

$${{{{{\rm{Hemolysis\; rate}}}}}}=\frac{{D}_{t}-{D}_{{nc}}}{{D}_{{pc}}-{D}_{{nc}}}\times 100\%$$

where *D*_t_, *D*_nc_, and *D*_pc_ were the absorbance of the tested sample, the negative control, and the positive control, respectively.

### Longitudinal relaxivity measurements

The longitudinal relaxivity measurements were performed on a 1.5 T clinical MRI instrument (iSpace Pro 1.5 T, Beijing Wandong Medical Technology Co., Ltd., Beijing, China). A series of aqueous solutions of PAA-Gd and Gd-DPTA were prepared in 2.0 mL Eppendorf tubes. The parameters for *T*_1_-weighted imaging were set as follows: echo time (TE) = 12 ms; repetition time (TR) = 500 ms; number of excitations (NEX) = 8.

The parameters for *T*_1_ value were measured by a fast gradient recalled echo sequence.

### Rodent animal studies

Rodent animals including BALB/c mice (6-week-old), C57 mice (6-week-old), and Sprague Dawley (SD) rats of the desired age were purchased from Vital River Animal Laboratories. Mice and rats were co-housed respectively and maintained on a 12-hour light–dark cycle with free access to food and autoclaved water in an air-conditioned SPF level animal room (22 ± 1 °C, 50–60% humidity, 4 mice/cage, 2 rat/cage).

### Establishment of rodent models

#### Rat model of right middle cerebral artery occlusion (rMCAO)

Male adult SD rats (~300 g) were used to construct the rMCAO model. Typically, the rats were in abrosia for 12 h besides water. Abdominal anesthesia was conducted with 1% pentobarbital. During surgery, the rectal temperature was monitored and kept at 37 ± 0.5 °C using a heating blanket. A midline incision between the sternum and chin was made to expose the carotid arteries. A MCAO suture (total length of ~45 mm and diameter of 0.38 ± 0.02 mm) with a slightly enlarged silicon-coated tip was inserted via external carotid artery into internal carotid artery and pushed ≈18–20 mm from the carotid bifurcation for building the rMCAO model. After that the skin of rat was sutured, the body temperature was maintained at 37 ± 0.5 °C using a heating pad until the rat woke up. The rat was subjected to MR scanning after 24 h.

#### Mouse models of vulnerable atherosclerotic plaques

ApoE^−/−^ C57 mice (6-week-old female) with a high-fat diet (containing 15% fat and 0.25% cholesterol) were purchased, and kept on a high-fat diet for another ~10 weeks. To ensure the formation of atherosclerotic plaques, the mice were subjected to MRI diagnosis every 4 weeks. The mice with plaques were selected for the angiography experiment.

#### Mouse model of carotid artery thrombosis

BALB/c mice (6-week-old male) were used to construct the carotid artery thrombosis model. Abdominal anesthesia was conducted with 1% pentobarbital. After the mouse were anesthetized, a midline incision between the sternum and chin was made to expose the carotid arteries. A ~3 mm trip of filter paper immersed in 10% FeCl_3_ was placed on the top of right carotid artery immediately. Three min later, the filter paper was removed and the injured area was rinsed three times with PBS to wash off the residual Fe^3+^. Thereafter, the skin of mouse was sutured, and the carotid thrombosis model of mouse was successfully constructed. The body temperature was maintained at 37 ± 0.5 °C using a heating pad until the mice woke up.

#### Rat model of CRF

The CRF rat model was established by the oral administration of adenine. In detail, SD rat (7-week-old male, 220-250 g) were administrated with natural saline suspension of adenine with a dose of 200 mg/kg every day through gastric gavage. Adenine is metabolized to 2,8-dihydroxyadenine, which crystallizes in tubular fluid, leading to chronic tubulointerstitial injury, renal insufficiency, and metabolic abnormalities characteristic of CRF. The body weights of rats were recorded every week to evaluate the degree of renal impairment. The CRF rat models would be established after ~30 days of adenine administration.

### In vivo MRI of rodent animals

All MR images of rodent animals were acquired using a 7 T animal MRI instrument (Bruker BioSpec 70/20).

The healthy rodent animals or the aforementioned disease models of rodent animals were anesthetized with 1-2% isoflurane delivered via a nose cone during the imaging sessions, and either the PAA-Gd or Gd-DTPA (0.1 mmol Gd per kilogram body weight) were intravenously injected through the tail vein. The different MRI sequences including 3D DCE angiography, TOF angiography, and *T*_1_-weighted imaging at different time point pre- and post-injection were selectively acquired through corresponding coil according to the demand.

### Histology study after MRI

The tissues of bilateral carotid arteries were harvested after MRI experiments of vulnerable atherosclerotic plaques and carotid artery thrombosis, and were fixed in the paraformaldehyde. After being embedded into paraffin, the fixed tissues were sliced. Then the resultant slices were stained with H&E, and subjected to microscopy study for the histological analysis. For the plaque imaging experiment, the adjacent slice next to H&E slice was further stained with α-SMA through immunohistochemical method, for judging the stability of plaque.

### Blood half-life measurement

PAA-Gd or Gd-DTPA contrast agents were intravenously injected into two groups of 6-week-old male BALB/c mice (*n* = 4), respectively. The dose level was the same as that for imaging experiments. Blood samples were obtained and weighted at 30 s, 5 min, 15 min, 30 min, 1 h, 2 h, 4 h, 8 h, and 24 h post injection. The Gd content in the blood was determined through ICP-MS after acid digestion.

### Body clearance pathway of PAA-Gd

PAA-Gd contrast agents were intravenously injected into 6-week-old male BALB/c mice (*n* = 4). The dose level was the same as that for imaging experiments. The urine and feces were continuously collected from the mice housed in the metabolic cage at different time points post-injection. Then these excretions were weighted and eroded to determine the Gd contents through ICP-MS for studying the PAA-Gd clearance. After 24 h, one mouse of was sacrificed and the major organs were extracted and subjected to H&E staining and CPN III staining. A healthy mouse was set as the control.

### Biosafety evaluation of PAA-Gd in healthy mice

A total of 12 healthy BALB/c mice (6-week-old female) were randomly divided into three groups (*n* = 4). Two groups of mice were intravenously injected with PAA-Gd contrast agent with a dose of 0.1 or 0.3 mmol Gd per kg body weight. The third group of mice was set as the control. The body weights of mice were recorded every day. At 14 d post-injection, the mice were sacrificed, and the major organs including the heart, liver, spleen, lung, and kidney were extracted, fixed with paraformaldehyde, cut into slices, and subjected to the histochemical analysis. The blood samples collected from three mice of each group were further used for blood routine and blood chemical examinations.

### Biosafety evaluation of PAA-Gd in healthy and CRF rats

A total of 12 CRF rats were randomly divided into three groups (*n* = 4). PAA-Gd and Gd-DTPA with a dose of 0.1 mmol Gd per kg body weight were intravenously injected into the two groups of rats, respectively. The third group of rats was set as the controls without any injection. In addition, 8 healthy rats with similar body weights (220 – 250 g) were randomly divided into 2 groups (*n* = 4), in which one group of rats were intravenously injected with PAA-Gd at the dose of 0.1 mmol Gd per kg body weight. The body weights of these rats were recorded every 2 d. The blood samples were collected at pre- and 7, 14, 21, 28 d post-injection of contrast agents and subjected to renal function-related-blood indexes examinations. After 28 d injection, the rats were sacrificed, and their kidneys were extracted, fixed with paraformaldehyde, cut into slices, and subjected to the H&E.

### In vivo MRI of swine

MRI of swine was acquired using a 3 T human MRI instrument (GE SIGNA PET/MR).

The young healthy Bama swines (two-month male) were purchased from Sichuan Greentech Bioscience Co., Ltd. After anesthetized with Zoletil® 50 delivered via intramuscular injection (3 mg/kg for the first time and 3 mg/kg for maintaining anesthesia), the swine were subjected to the imaging equipment, and the PAA-Gd (0.1 or 0.03 mmol Gd per kilogram body weight) or Gd-DTPA (0.1 mmol Gd per kilogram body weight) were intravenously injected into the swine through small saphenous vein o3f hind leg. The human head coil was adopted for imaging. The different MRI sequences including 3D-TRICKS, 3D BRAVO, Oblique Axis TOF MRA, and Sagittal 3D PC MRA, were adopted for imaging at designed time points pre- and post-injection.

### Biosafety evaluation of PAA-Gd in swine

Following the MRI, the young swine with an injection of PAA-Gd (0.03 mmol Gd per kg body weight) was continued to be cultivated. In addition, another sexually matured swine (4-month male, purchased from Sichuan Greentech Bioscience Co., Ltd.) was adopted, and the PAA-Gd with the dose of 0.1 mmol Gd per kg body weight was intravenously injected. The rectal temperatures and body weights of swines were recorded every day. The blood samples were collected from the front cavity vein of swine at pre- and 7, 14, 21, 28 d post-injection of PAA-Gd and subjected for blood routine and blood chemical examinations. After one month injection, the swines were sacrificed, and the major organs including heart, liver, spleen, lung, kidney, brain, and bladder were extracted, fixed with paraformaldehyde, cut into slices, and subjected to the H&E and CPN III staining.

All animal experiments were performed according to a protocol approved by the Peking University Institutional Animal Care and Use Committee (LA2019083).

### Main parameters of MRI studies

The main parameters of MRI studies in the current work were set as follows:

#### 1.5 T MRI

The detailed parameters for *T*_1_-weighted imaging were set as follows: echo time (TE) = 12 ms; repetition time (TR) = 500 ms; number of excitations (NEX) = 8.

*T*_1_ relaxation time was obtained through 2D-Fast Gradient Recalled Echo sequence with multi-flip angle. The detailed parameters: TE = 10 ms, TR = 80 ms, FA = 7, 10, 12, 15, 17, 20, 25, 30, 35, 40, 45, 50, 55, 60, 65, 70, 75, 80, 85, and 90°, NEX = 4.

The data was analyzed by MatLab (R2019b).

#### 7.0 T MRI

The detailed parameters for 3D DCE angiography of mouse head and thorax with mouse body coil were set as follows: TE = 1.466 ms, TR = 12 ms, FOV = 50 mm × 27 mm × 20 mm, MTX = 256 × 138 × 66, slice thickness = 0.303 mm.

The detailed parameters for 3D DCE angiography of rat head with rat head coil were set as follows: TE = 1.962 ms, TR = 12.927 ms, FOV = 40 mm × 40 mm × 35 mm, MTX = 400 × 400 × 100, slice thickness = 0.35 mm.

The detailed parameters for 2D TOF angiography of rats with head coil were set as follows: TE = 2.188 ms, TR = 12 ms, FOV = 40 mm × 35 mm, MTX = 400 × 350, slice thickness = 0.5 mm.

The detailed parameters for 3D DCE angiography of mouse head with mouse head coil were set as follows: TE = 1.41 ms, TR = 11.1 ms, FOV = 25 mm × 20 mm × 25 mm, MTX = 179 × 143 × 80, slice thickness = 0.313 mm.

The detailed parameters for 3D DCE angiography of mouse head with mouse head surface coil were set as follows: TE = 1.41 ms, TR = 11.1 ms, FOV = 25 mm × 20 mm × 25 mm, MTX = 179 × 143 × 80, slice thickness = 0.313 mm.

The detailed parameters for 3D DCE angiography of mouse liver and kidney with mouse body coil were set as follows: TE = 1.4 ms, TR = 11 ms, FOV = 35 mm × 35 mm × 25 mm, MTX = 180 × 150 × 80, slice thickness = 0.313 mm.

The detailed parameters for 3D DCE angiography of mouse atherosclerotic plaque with mouse head coil were set as follows: TE = 1.4 ms, TR = 11 ms, FOV = 30 mm × 25 mm × 25 mm, MTX = 180 × 150 × 80, slice thickness = 0.313 mm.

The detailed parameters for *T*_1_-weighted imaging of mouse atherosclerotic plaque with mouse head coil were set as follows: TE = 5.34 ms, TR = 810 ms, FOV = 19 mm × 19 mm, MTX = 190 × 190, slice thickness = 0.5 mm.

The detailed parameters for 3D DCE angiography of mouse thrombosis and thrombolytic therapy with mouse head coil were set as follows: TE = 1.4 ms, TR = 11 ms, FOV = 25 mm × 25 mm × 25 mm, MTX = 167 × 167 × 80, slice thickness = 0.313 mm.

The detailed parameters for 2D TOF angiography of mouse thrombosis with mouse head coil were set as follows: TE = 1.5 ms, TR = 12 ms, FOV = 19 mm × 19 mm, MTX = 127 × 127, slice thickness = 1 mm.

#### 3.0 T MRI

The detailed parameters for 3D-TRICKS were set as follows: TE = Minimum, TR = 3.3 ms, FOV = 320 mm × 288 mm, MTX = 256 × 224, slice thickness = 1.6 mm.

The detailed parameters for 3D BRAVO were set as follows: TE = 3 ms, and TR = 8 ms, FOV = 240 mm × 216 mm, MTX = 256 × 256, slice thickness = 1.0 mm.

The detailed parameters for TOF MRA were set as follows: TE = Minimum, TR = Minimum, FOV = 240 mm × 220 mm, MTX = 416 × 224, slice thickness = 1.2 mm.

The detailed parameters for PC MRA were set as follows: TE = Minimum, and TR = Minimum, FOV = 280 mm × 252 mm, MTX = 320 × 256, slice thickness = 1.4 mm.

### Statistical analysis

Data are expressed as mean ± standard deviation (SD) as indicated in the figure captions. Statistical differences of two groups were determined by T-test. A *p* value of <0.05 was regarded as statistically different. All tests were carried out by GraphPad Prism software (v7.0 & v8.0), and the charts were drawn by OriginPro (9.0 & 2019b) and GraphPad Prism software (v8.0).

### Reporting summary

Further information on research design is available in the Nature Portfolio Reporting Summary linked to this article.

### Supplementary information

Supplementary Information File

Description of Additional Supplementary Files

Supplementary video 1

Supplementary video 2

Supplementary video 3

Supplementary video 4

Supplementary video 5

Supplementary video 6

Supplementary video 7

Supplementary video 8

Supplementary video 9

Supplementary video 10

Supplementary video 11

Reporting Summary

### Source data

Source Data

## Data Availability

The main data supporting the findings of this study are available in the article and the Supplementary Information file. The raw datasets generated during the study are not publicly shared but are available for research purposes from the corresponding author upon request. Source data are provided in this paper.
