# Supplementary material for: Hypersensitive MR angiography based on interlocking stratagem for diagnosis of cardiac-cerebral vascular diseases
Source: Nat Commun. 2023 Oct 2;14:6149. doi: 10.1038/s41467-023-41783-9 (PMC10545789; doi:10.1038/s41467-023-41783-9)
Supplement: Supplementary file 1 — Supplementary Information File [file 41467_2023_41783_MOESM1_ESM.pdf]

Supplementary Information

Hypersensitive MR Angiography based on Interlocking Stratagem for Diagnosis of Cardiac-cerebral Vascular Diseases

Peisen Zhang[1,#], Junwei Cheng[1,#], Yijie Lu[2,#], Ni Zhang[3], Xiaoai Wu[3], Hua Lin[3], Wei Li[4,*], Jian Wang[5], Mitchell A. Winnik[2], Zhihua Gan[1,*], Yi Hou[1,*]

[1] College of Life Science and Technology, Beijing University of Chemical Technology, Beijing 100029, China.

[2] Department of Chemistry, University of Toronto, Toronto, Ontario M5S 3H6, Canada

[3] Department of Psychiatry, and Department of Nuclear Medicine, West China Hospital, Sichuan University, Chengdu 610041, China

[4] Department of Nanomedicine & International Joint Cancer Institute, Naval Medical University, Shanghai 200433, China

[5] Department of Head and Neck Surgery, National Cancer Center/National Clinical Research Center for Cancer/Cancer Hospital, Chinese Academy of Medical Sciences, Peking Union Medical College, Beijing 100021, China

[#]These authors contributed equally to this work.

*To whom correspondence should be addressed. E-mail: houyi@iccas.ac.cn; zhgan@mail.buct.edu.cn; liwei_dds@163.com.

Supplementary Note 1. Synthesis and Characterization of PAA-Gd

The synthetic route of zwitterionic MCP contrast agent PAA-Gd is illustrated in Figure S1. DTPA was covalently linked to PAA through the DET linker *via* amidation reaction, which was catalyzed by DMTMM. The PAA-Gd contrast agent was obtained through the chelating reaction between residual carboxyl groups of DTPA and Gd^{3+} ions.

The ^{1}H NMR spectra of PAA, PAA-DET and PAA-DTPA in each synthesis step (carried out on a 400 MHz Nuclear Magnetic Resonance Spectrometer (AVANCE III)) are displayed in the Figure S2-S4 (analyzed by MestReNova software (9.0.1.13254)). The proton signals of methylene and methylidyne on the backbone of PAA (a & b) appeared at 1.40 and 2.02 ppm, respectively. After conjugation with diethylenetriamine (DET) and DTPA in turn, the proton signals of the methylene of DET (c, d, e and f), and the proton signals of the methylene of DTPA (g, h, i and j) appeared within the range of 2.50-4.10 ppm. The proton signals of the backbone (a & b) moved slightly to a higher magnetic field, indicating that DET and DTPA were successfully combined with the main chain of PAA.

According to the integrated peaks areas, ~92.0% of carboxyl groups in PAA were convert to amino groups after conjugating with DET, and *ca.* 86.0% of carboxyl groups were finally conjugated with DTPA. The amount of Gd^{3+} chelated by PAA-Gd was estimated through thermo gravimetric analysis (TGA), and the result suggests that almost all of DTPA groups (~99.7%) chelate Gd^{3+}, thus the Gd^{3+} content of the polymeric contrast agent is *ca.* 1.2 mmol/g MCP.

In addition, the polydispersity indices (PDI) of PAANa and PAA-DTPA measured by an aqueous gel-permeation chromatography (GPC) system (GPC Waters 1515) calibrated with poly(ethylene glycol) standards were 1.20 and 1.32, respectively, indicating that the samples were well dispersed and no inter- or intra-molecular cross-linking occurred during the synthesis process.

The impact of the above synthesis process on the properties of the polymer was further investigated with dynamic light scattering (DLS) carried out at a Nano Zetasizer (Malvern). The results in Figure 1c revealed that the hydrodynamic diameter (d_H) of

PAA molecules is reasonably increased from 4.0 nm to 4.9 nm after conjugation of DET linkers, and continue to increase to 5.1 nm after PAA-DTPA was synthesized. After Gd^{3+} chelation, the d_H of polymers eventually grew to 7.7 nm, implying that the PAA-Gd molecule is successfully constructed. Besides, the DLS profile remains nearly unchanged through the conjugation and coordination reaction except for the shift owing to the increased size of polymers, suggesting that the conjugation reaction took place in a controlled manner, which did not lead to unwanted aggregates.

In addition, the impact of the conjugation and coordination reaction on the electrophoretic mobility of the polymers were also investigated (Figure 1d). The electrophoretic mobility can be determined by the *Henry Equation*:

$$U_E = \frac{2\varepsilon z f(Ka)}{3\eta}$$

where U_E is the electrophoretic mobility, ε is the dielectric constant, z is the zeta potential, f(Ka) is Henry's function with value of 1.5 in PBS buffer, and η is the viscosity. In physiological PBS buffer (pH 7.4), the electrophoretic mobility of PAA polymers significantly increased from -1.26 $m^2V^{-1}s^{-1}10^{-8}$ to 0.64 $m^2V^{-1}s^{-1}10^{-8}$ during the DET conjugation, which suggested that the carboxyl groups in the side chains of PAA have been convert to amino groups. Thereafter, through the connection of DTPA, the electrophoretic mobility of polymers decreased to -1.37 $m^2V^{-1}s^{-1}10^{-8}$ due to the multiple carboxyl groups in the DTPA molecules. At last, through being coordinated with Gd^{3+}, the electrophoretic mobility of PAA-Gd molecule was re-increased to -0.24 $m^2V^{-1}s^{-1}10^{-8}$ owing to the positive charge carried by the Gd^{3+} ions, which is almost neutral in physiological conditions. This variation of electrophoretic mobility implied that after the above-mentioned multi-step reaction, the zwitterionic PAA-Gd molecules were successfully synthesized.

Supplementary Note 2. Calculation of the Elimination Half-life of Contrast Agent Through Two-compartment Model

In the two-compartment model, the decline of drug concentration can be divided into two distinct phases, i.e., the distribution phase with initial rapid decline in serum drug concentration, and the elimination phase with slow decline in drug concentration.

The clearance of drugs can be mathematically described as a bi-exponential function:

$$C(t) = Ae^{-\alpha t} + Be^{-\beta t}$$

Where t is the time since the administration of drug; C(t) is the drug concentration; A and B are coefficients that describe the exponential functions of distribution phase and elimination phase; α and β are exponents that describe the shape of the curve for distribution phase and elimination phase.

After the distribution of the drug achieve an equilibrium, the above function can be simplified as:

$$C(t) = Be^{-\beta t}$$

Therefore, the elimination half-life of the drug can be expressed as:

$$t_{1/2\beta} = \frac{ln2}{\beta}$$

Accordingly, through data fitting, the elimination half-life of the PAA-Gd and Gd-DTPA contrast agent can be calculated as 73.8 and 11.4 min, respectively.

3. Supplementary Figures

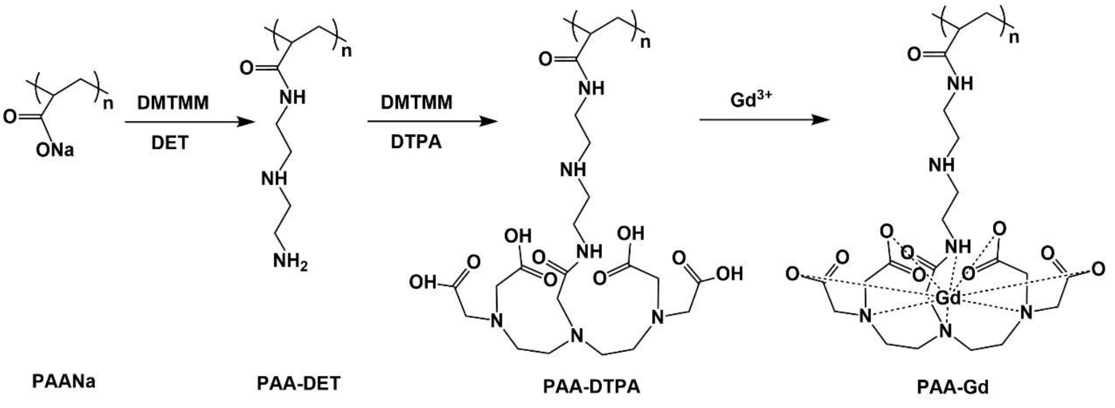

Figure S1 The synthetic route of zwitterionic PAA-Gd contrast agent.

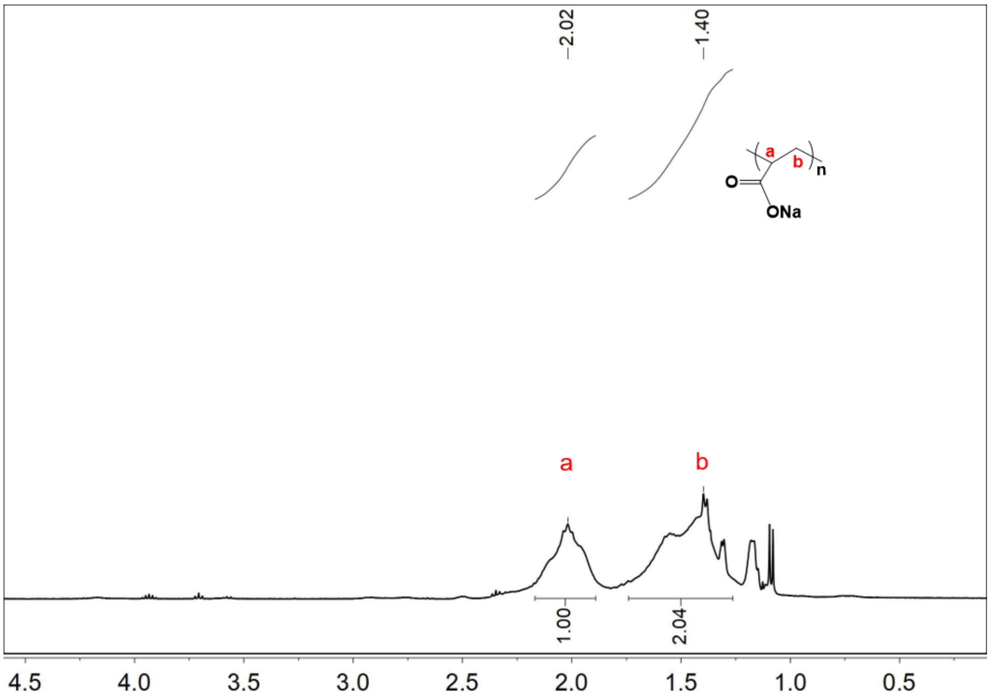

Figure S2 ^{1}H NMR Spectra (D_2O) of PAANa with the numbers of hydrogen atoms determined by integration of the peak areas.

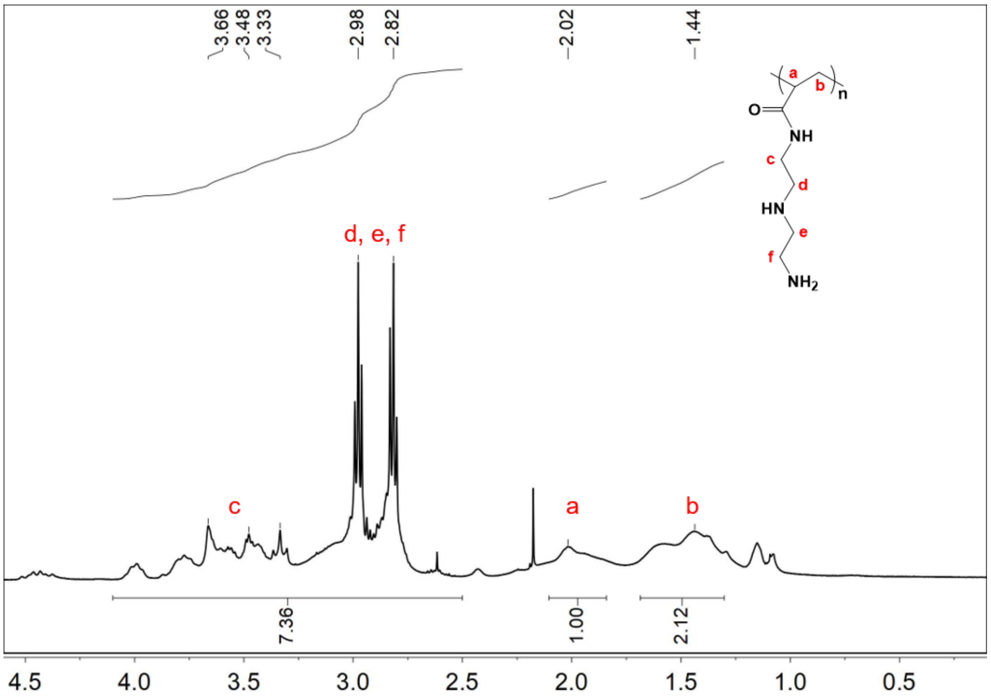

Figure S3 ^{1}H NMR Spectra (D_2O) of PAA-DET with the numbers of hydrogen atoms determined by integration of the peak areas. (Approximately 49 of the 54 carboxyl groups on the side chain of PAA molecule are attached to DET).

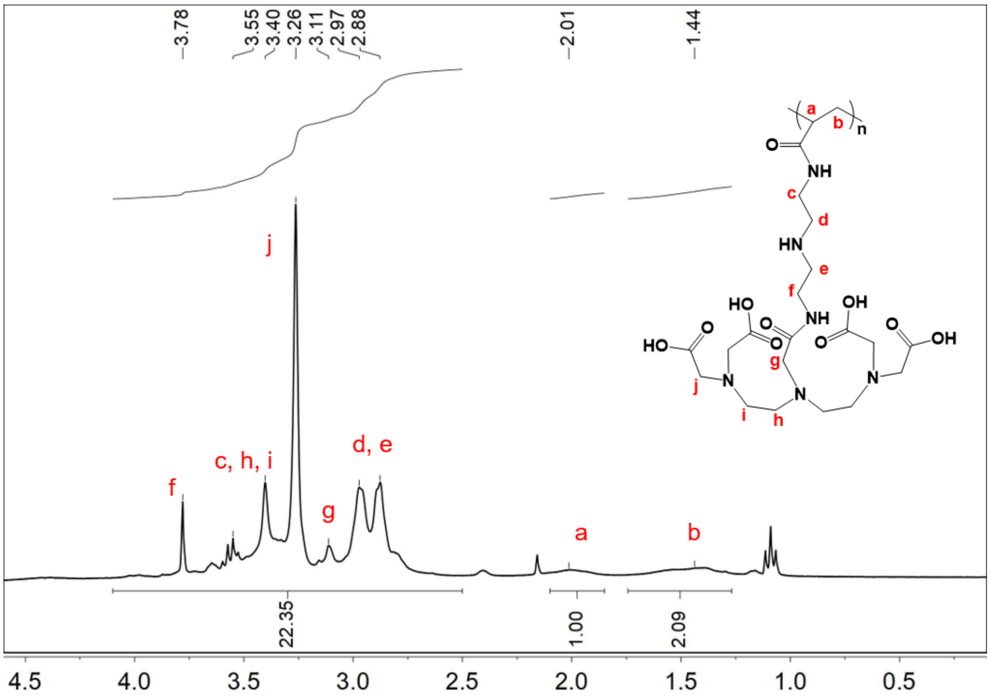

Figure S4 ^{1}H NMR Spectra (D_2O) of PAA-DTPA with the numbers of hydrogen atoms determined by integration of the peak areas. (Approximately 42 of the 49 $-NH_2$ of PAA-DET molecule were further coupled to DTPA).

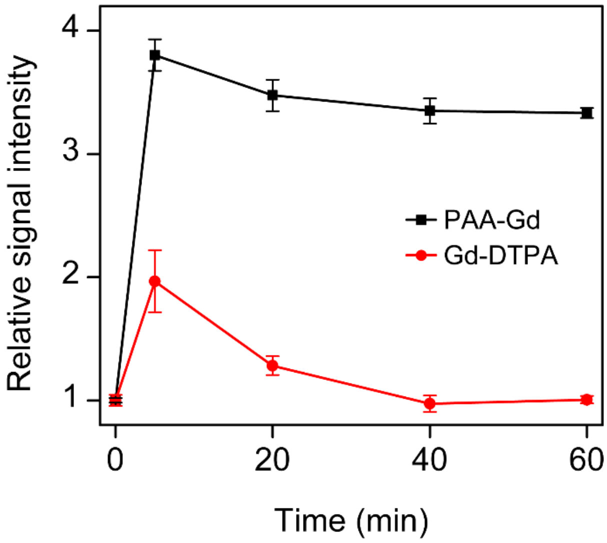

Figure S5 Temporal evolution of the average intravascular relative signal intensity before and at different time points after intravenous injection of PAA-Gd or Gd-DTPA, respectively (n = 3, data were plotted as mean ± standard deviation).

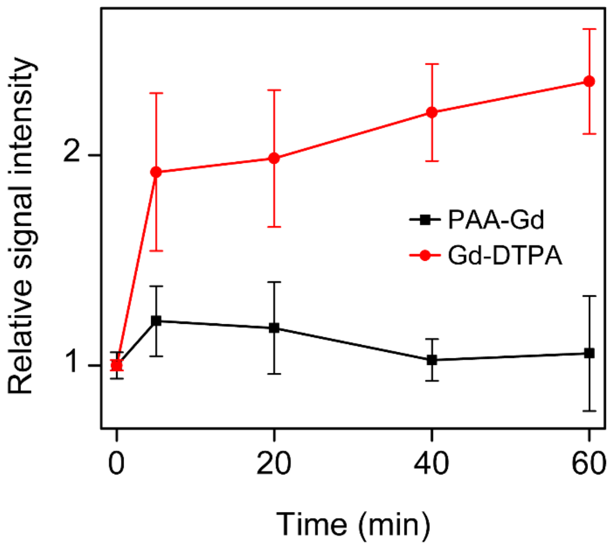

Figure S6 Temporal evolution of the average liver parenchyma relative signal intensity before and at different time points after intravenous injection of PAA-Gd or Gd-DTPA, respectively (n = 3, data were plotted as mean ± standard deviation).

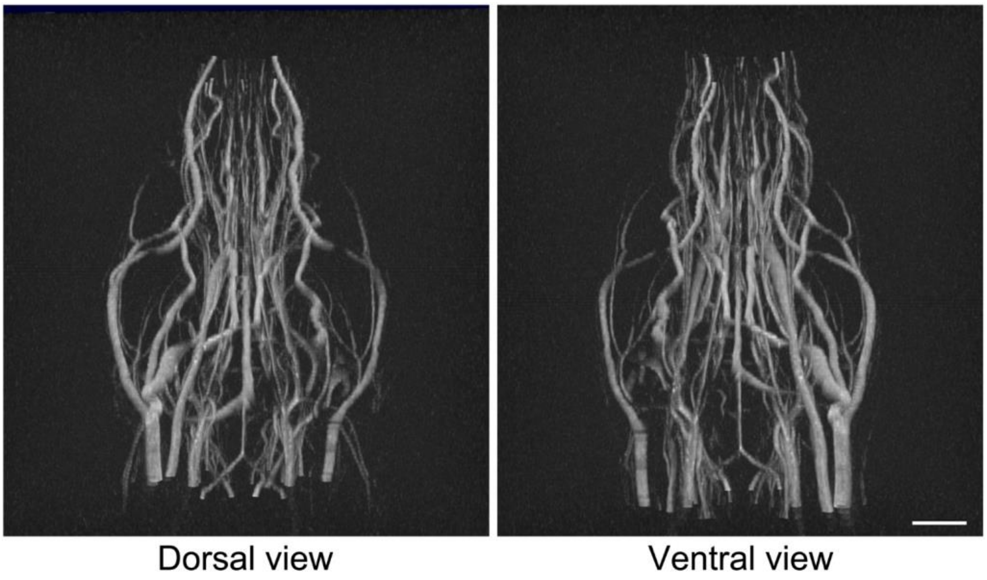

Figure S7 TOF MR angiography of rat head obtained before PAA-Gd injection. The embedded scale bar corresponded to 5 mm. Triplicates were performed independently with similar results.

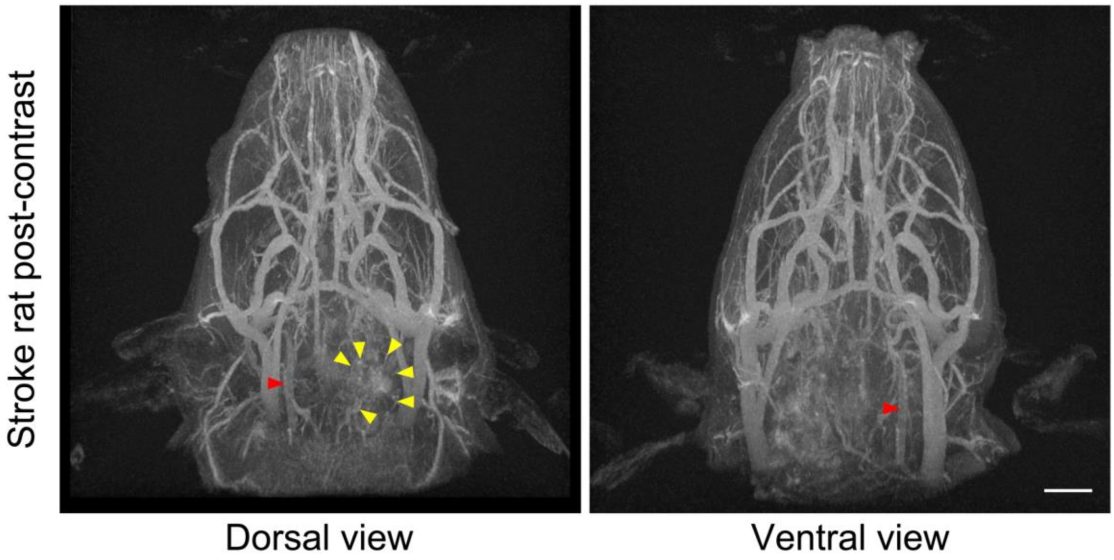

Figure S8 PAA-Gd-enhanced 3D MR angiography of the rat head after ischemic stroke, in which the left carotid artery and the compensatory angiogenesis surrounding the blocked right carotid artery were indicated by the red arrow and yellow arrows, respectively. The embedded scale bar corresponded to 5 mm. Triplicates were performed independently with similar results.

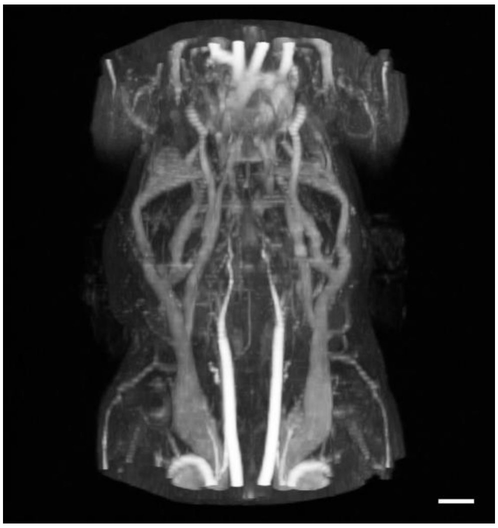

Figure S9 PAA-Gd-enhanced 3D MR angiography of the mouse head acquired through using conventional head coil. The embedded scale bar corresponded to 2 mm. Triplicates were performed independently with similar results.

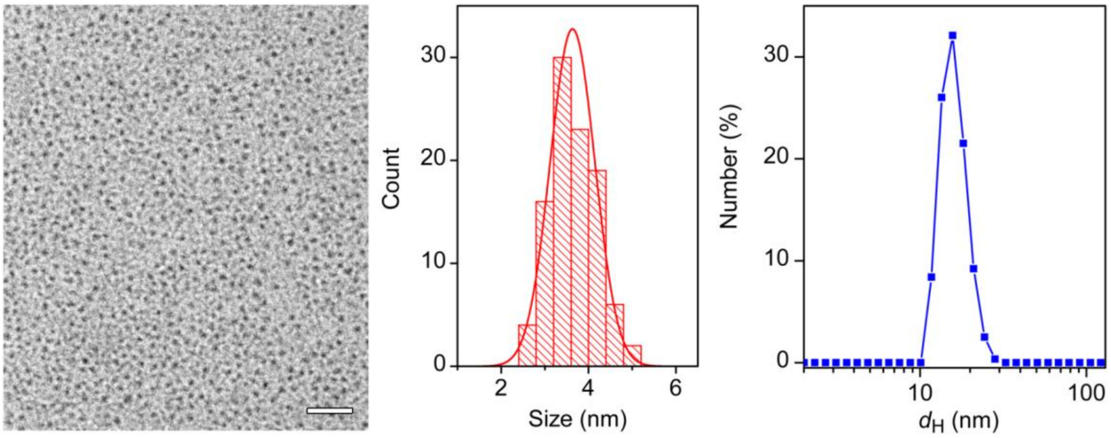

Figure S10 the transmission electron microscope (left) image, size distribution profiles (middle), and DLS result (right) of PEGylated $NaGdF_4$ nanoparticles. The embedded scale bar corresponded to 30 nm. At least five repetitions were performed independently with similar results. Transmission electron microscope image was conducted on a transmission electron microscope (JEOL, JEM-2100).

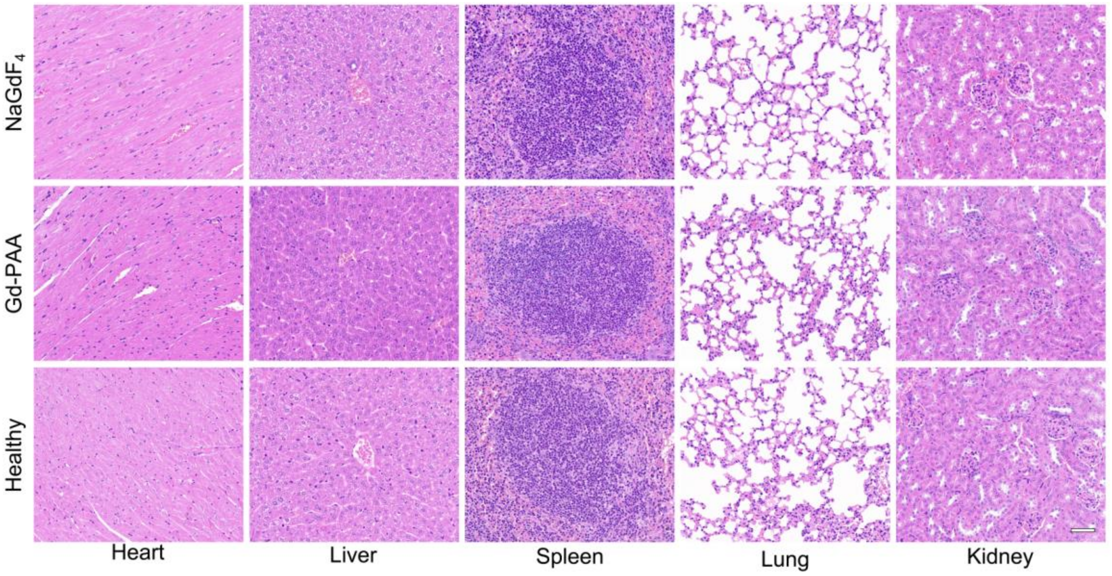

Figure S11 H&E staining of tissue slices of major organs from mice treated with different agents. The embedded scale bar corresponded to 50 µm. Triplicates were performed independently with similar results.

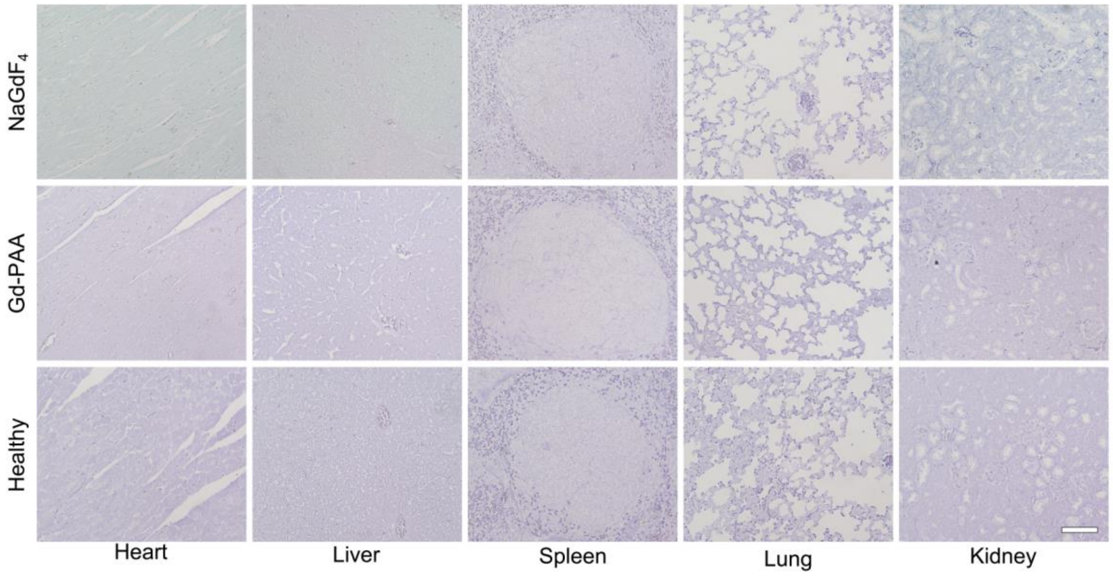

Figure S12 CPNIII staining of tissue slices of major organs from mice treated with different agents. The embedded scale bar corresponded to 50 μm. Triplicates were performed independently with similar results.

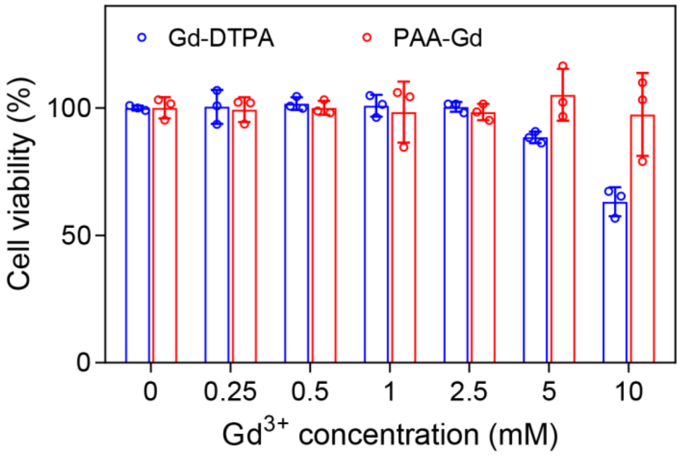

Figure S13 Cell viability assay of PAA-Gd and Gd-DTPA contrast agent (n = 3, data were plotted as mean ± standard deviation).

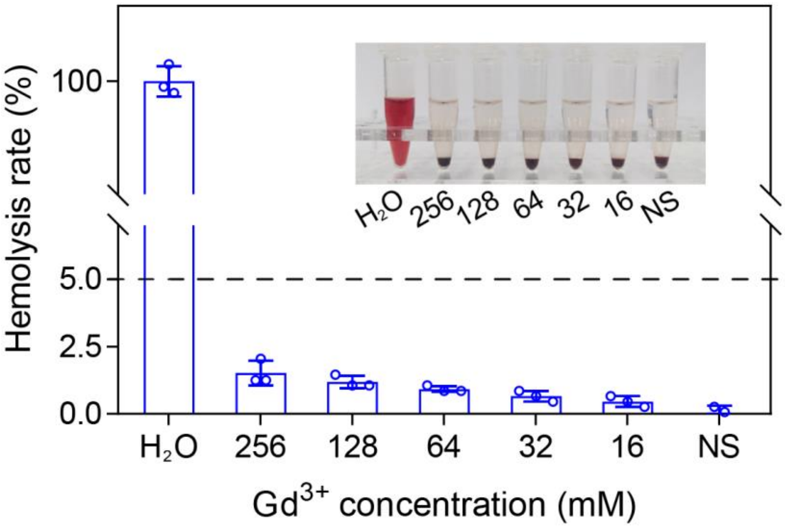

Figure S14 Hemolysis rates of PAA-Gd contrast agent at different Gd^{3+} concentrations (n = 3, data were plotted as mean ± standard deviation).

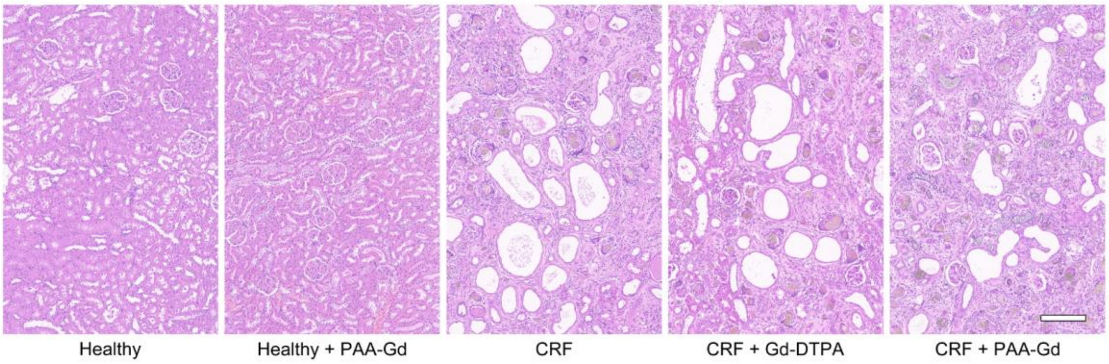

Figure S15 H&E staining of kidney tissues from the representative rats in each group. The embedded scale bar corresponds to 200 µm. Triplicates were performed independently with similar results.

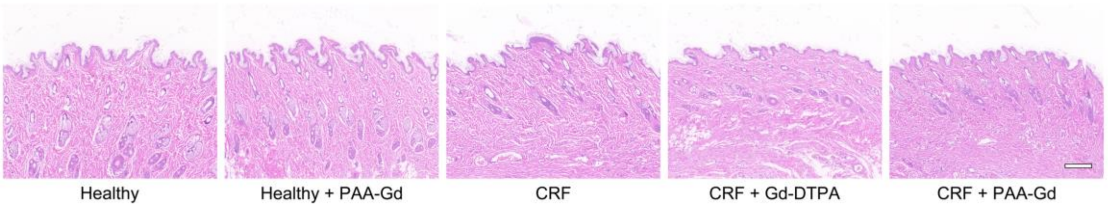

Figure S16 H&E staining of skin tissues from the representative rats in each group. The embedded scale bar corresponds to 200 µm. Triplicates were performed independently with similar results.

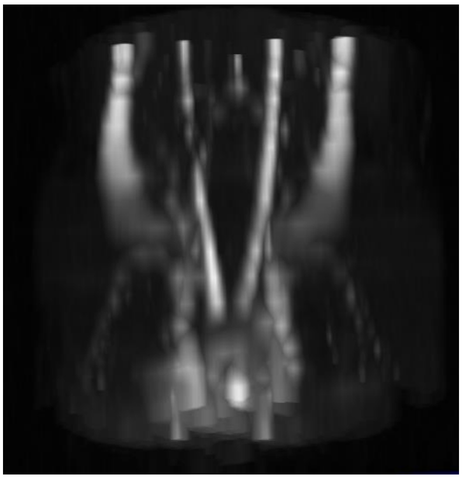

Figure S17 TOF MR angiography of the $FeCl_3$-induced carotid arterial thrombosis mouse model.

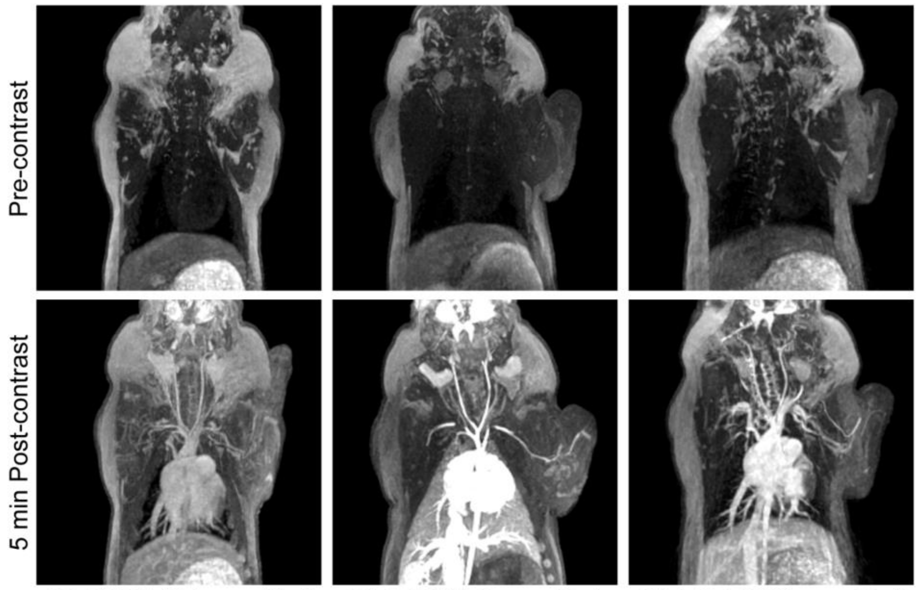

Figure S18 3D TRICKS of swine acquired pre- and post-injection of Gd-DTPA at the dose of 0.1 mmol per kg weight and PAA-Gd at the dose of 0.1 mmol/kg or 0.03 mmol/kg, respectively.

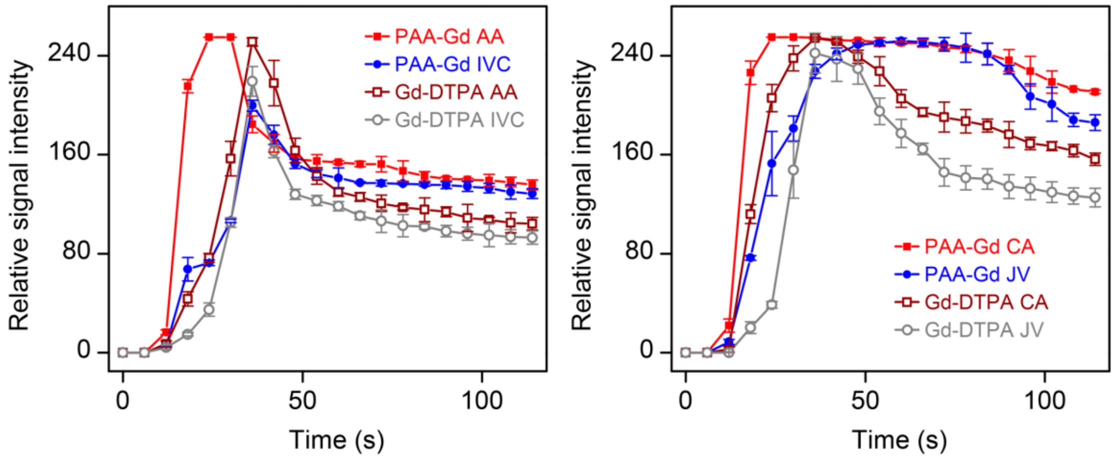

Figure S19 Temporal evolution of the average intravascular MRI signal intensity before and at different time points after intravenous injection of PAA-Gd (0.03 mmol/kg) or Gd-DTPA (0.1 mmol/kg), respectively (n = 3, data were plotted as mean ± standard deviation).

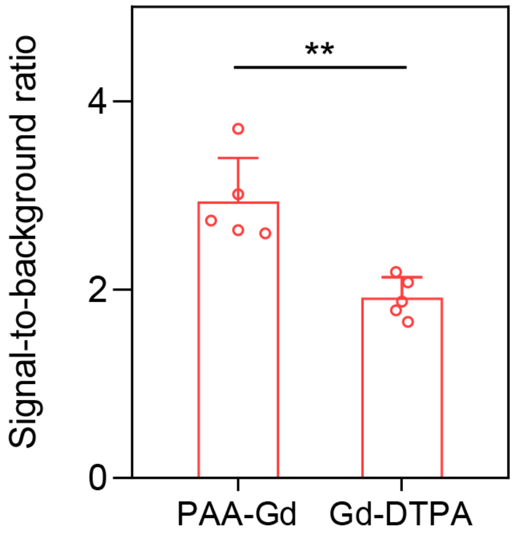

Figure S20 The vascular signal-to-background ratio between five different blood vessels and their surrounding normal tissues (n = 5) of swine after injecting with PAA-Gd or Gd-DTPA, respectively, together with their statistical difference. Statistical significance was determined by two-sided unpaired T-test, $p = 0.02$ (**$p < 0.01$). Data were plotted as mean ± standard deviation.

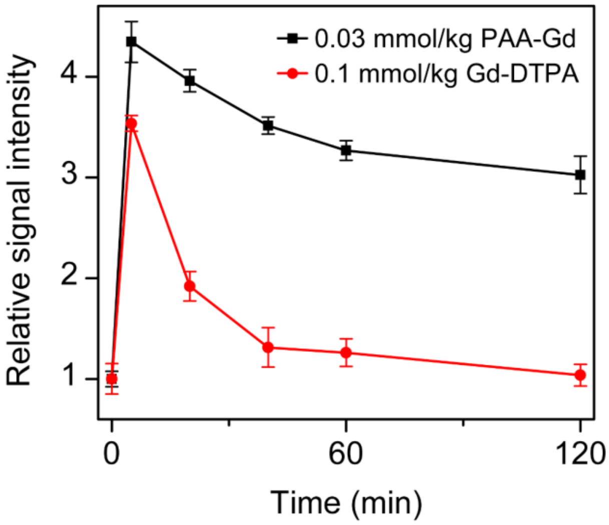

Figure S21 Temporal evolution of the average intravascular T_1 signal intensity before and at different time points after intravenous injection of PAA-Gd or Gd-DTPA, respectively (n = 3, data were plotted as mean ± standard deviation).

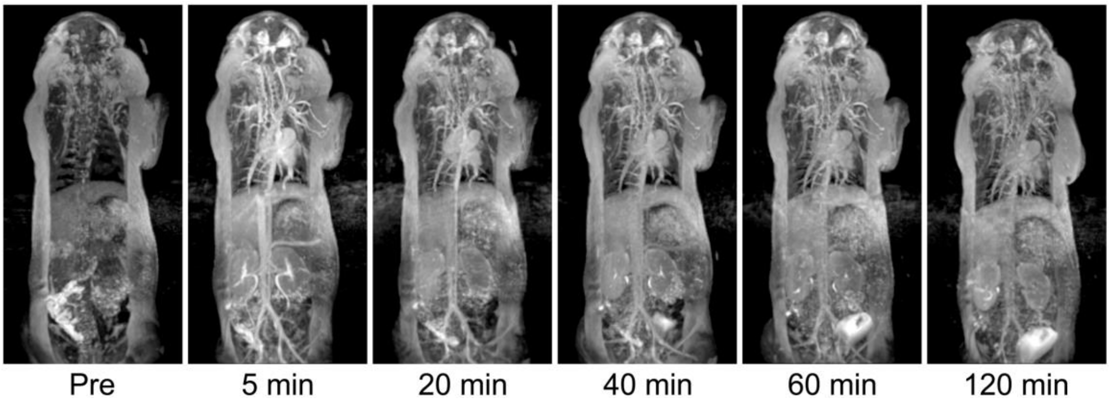

Figure S22 Delayed 3D BRAVO T_1-weighted MRI obtained at different time points post-injection of PAA-Gd with 0.03 mmol/kg.

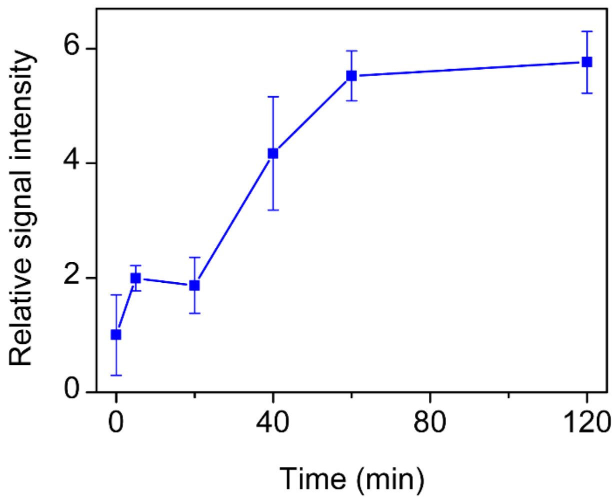

Figure S23 Temporal evolution of the T_1 relative signal intensity of the bladder region (n = 3).

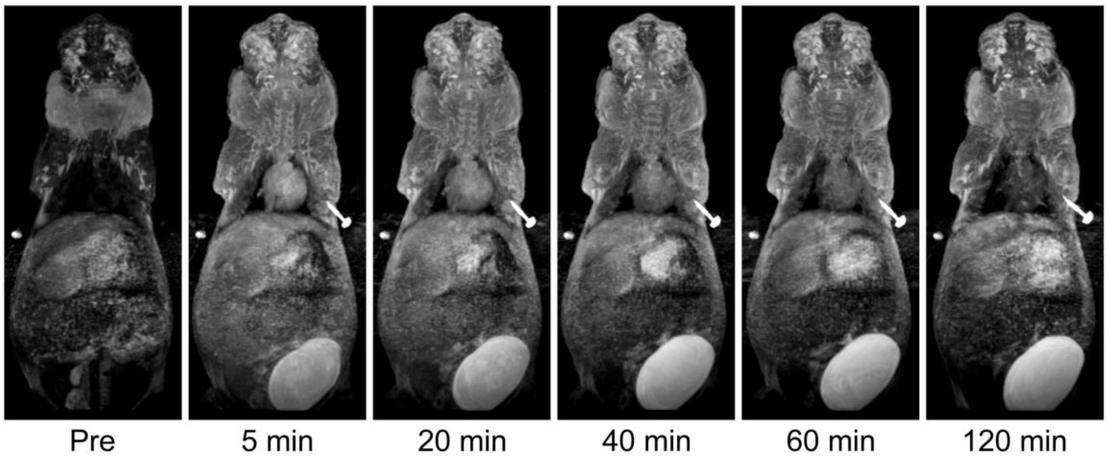

Figure S24 Delayed 3D BRAVO T_1-weighted MRI obtained at different time points post-injection of Gd-DTPA with 0.1 mmol/kg.

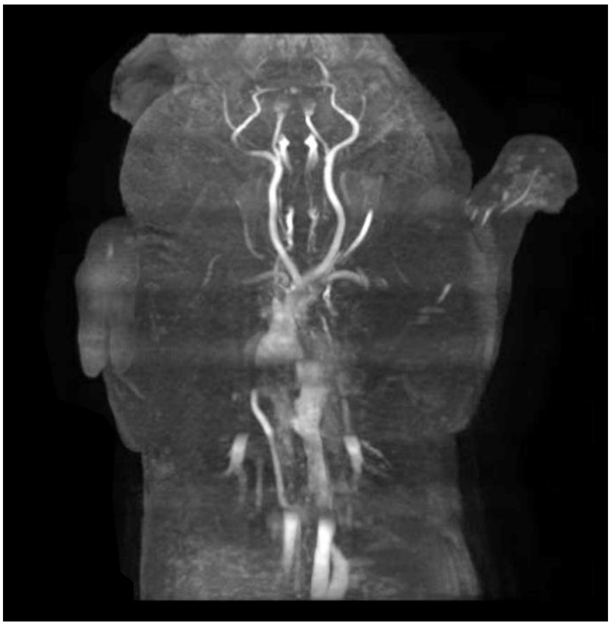

Figure S25 3D TOF MR angiography of swine.

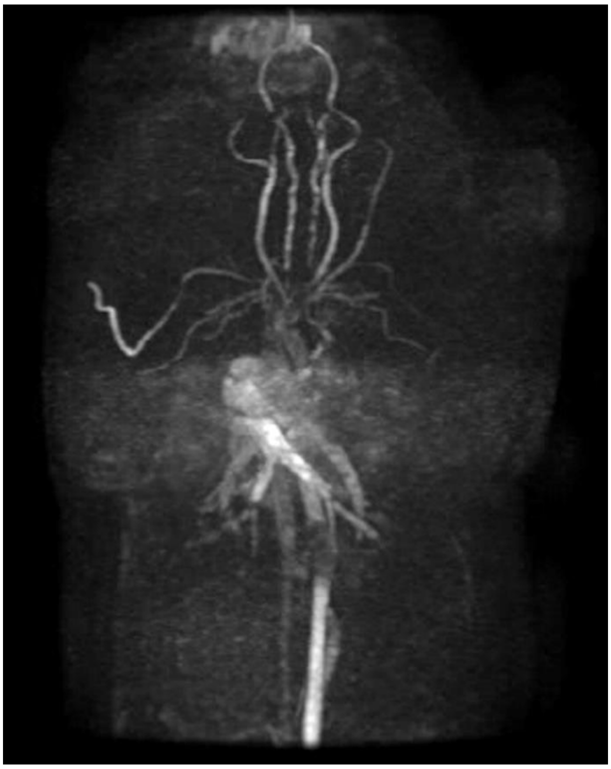

Figure S26 3D PC MR angiography of swine.

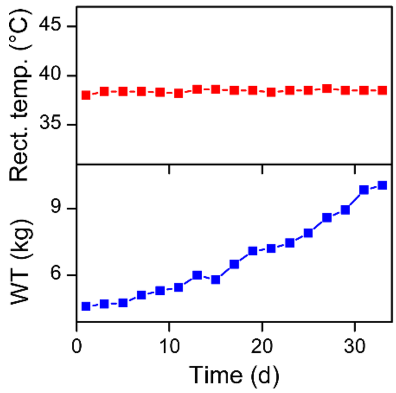

Figure S27 Fluctuations in the rectal temperature and the increase of body weight of young swine after PAA-Gd administration.

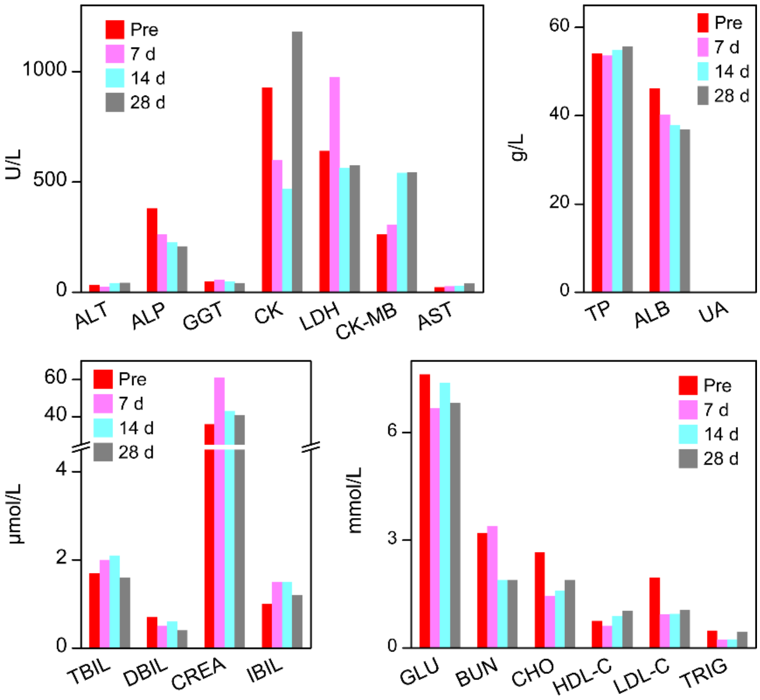

Figure S28 Blood biochemical test results of young swine treated with the PAA-Gd contrast agents.

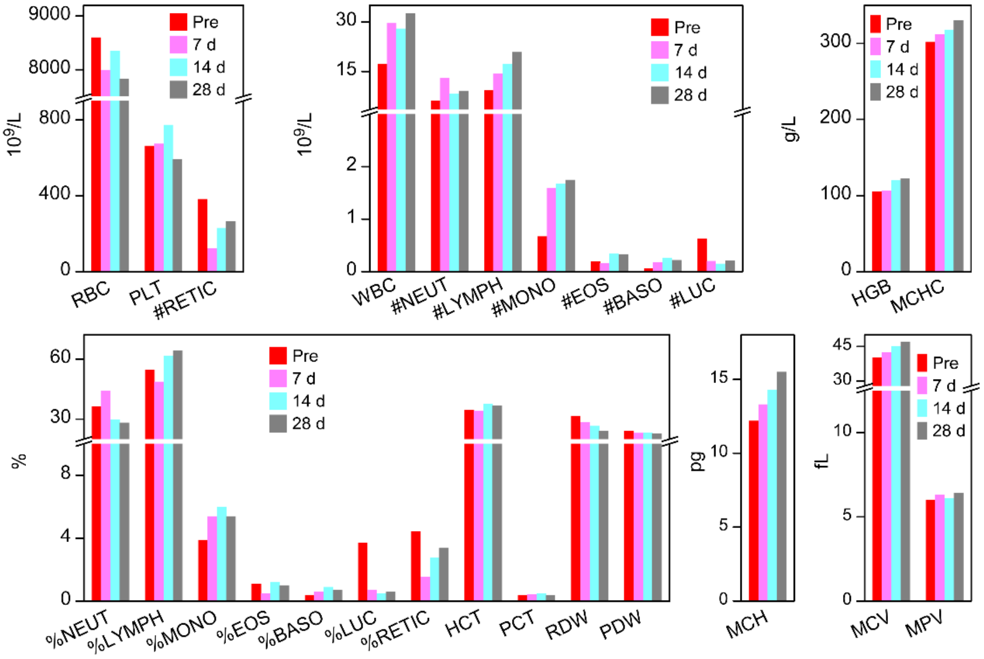

Figure S29 Routine blood test results of young swine treated with the PAA-Gd contrast agents.

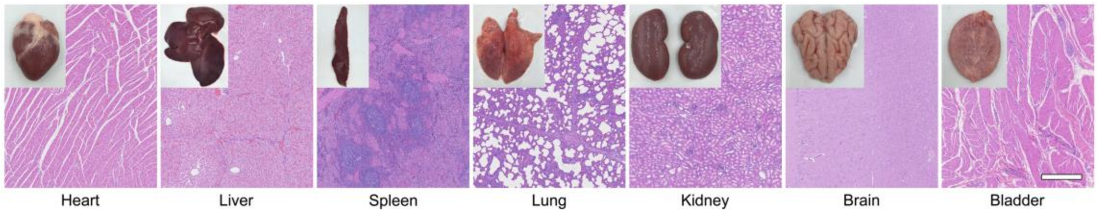

Figure S30 H&E staining of tissue slices from major organs of young swine treated with the PAA-Gd at 33 d post-injection. The embedded scale bar corresponded to 500 μm. Triplicates were performed independently with similar results.

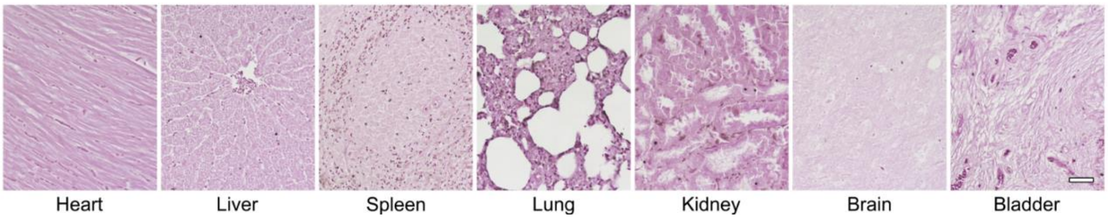

Figure S31 CPN III staining of tissue slices of major organs from swine injected with PAA-Gd (Gd 0.1 mmol/kg). The embedded scale bar corresponded to 50 μm. Triplicates were performed independently with similar results.

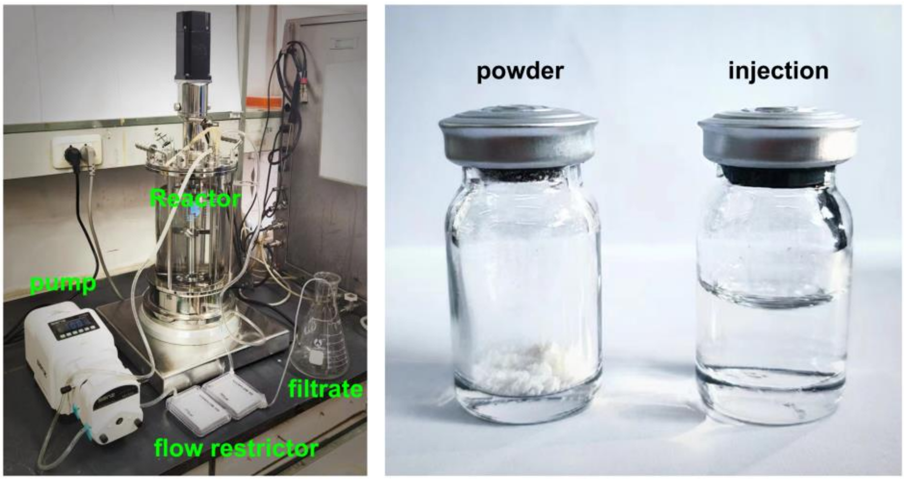

Figure S32 The digital photograph of practical equipment of the large-scaled PAA-Gd synthesis system (left) and the standardly packaged PAA-Gd powder and injection for the potential clinical use (right).
