# Supplementary material for: Hypersensitive MR angiography based on interlocking stratagem for diagnosis of cardiac-cerebral vascular diseases
Source: Nat Commun. 2023 Oct 2;14:6149. doi: 10.1038/s41467-023-41783-9 (PMC10545789; doi:10.1038/s41467-023-41783-9)
Supplement: Supplementary file 2 — Description of Additional Supplementary Files [file 41467_2023_41783_MOESM2_ESM.pdf]

Title: Supplementary video 1
Description: 3D DCE MR angiography of the head and thorax of the health mouse before intravenous injection of PAA-Gd.

Title: Supplementary video 2
Description: 3D DCE MR angiography of the head and thorax of the health mouse after intravenous injection of PAA-Gd.

Title: Supplementary video 3
Description: 3D DCE MR angiography of the head and thorax of the health mouse before intravenous injection of Gd-DTPA.

Title: Supplementary video 4
Description: 3D DCE MR angiography of the head and thorax of the health mouse after intravenous injection of Gd-DTPA.

Title: Supplementary video 5
Description: Axial TOF angiography show of the rat head.

Title: Supplementary video 6
Description: 3D DCE MR angiography of the rat head before intravenous injection of PAA-Gd.

Title: Supplementary video 7
Description: 3D DCE MR angiography of the rat head after intravenous injection of PAA-Gd.

Title: Supplementary video 8
Description: 3D hepatorenal MR angiography of mouse obtained at 30 min post-injection of PAA-Gd.

Title: Supplementary video 9
Description: PAA-Gd enhanced 3D angiography of mouse carotid thrombosis before treatment.

Title: Supplementary video 10
Description: The spatial evolution of blood vessels of swine derived from 3D BRAVO imaging (slice by slice).

Title: Supplementary video 11
Description: The spatial evolution of blood vessels of swine derived from 3D BRAVO imaging (from ventral to dorsal level).
