# Supplementary material for: Hypersensitive MR angiography based on interlocking stratagem for diagnosis of cardiac-cerebral vascular diseases
Source: Nat Commun. 2023 Oct 2;14:6149. doi: 10.1038/s41467-023-41783-9 (PMC10545789; doi:10.1038/s41467-023-41783-9)
Supplement: Supplementary file 14 — Reporting Summary [file 41467_2023_41783_MOESM14_ESM.pdf]

nature portfolio

Corresponding author(s): Yi Hou

Last updated by author(s): Aug 6, 2023

Reporting Summary

Nature Portfolio wishes to improve the reproducibility of the work that we publish. This form provides structure for consistency and transparency in reporting. For further information on Nature Portfolio policies, see our Editorial Policies and the Editorial Policy Checklist.

Statistics

For all statistical analyses, confirm that the following items are present in the figure legend, table legend, main text, or Methods section.

n/a	Confirmed	
☐	☒	The exact sample size (*n*) for each experimental group/condition, given as a discrete number and unit of measurement
☐	☒	A statement on whether measurements were taken from distinct samples or whether the same sample was measured repeatedly
☐	☒	The statistical test(s) used AND whether they are one- or two-sided<br>*Only common tests should be described solely by name; describe more complex techniques in the Methods section.*
☒	☐	A description of all covariates tested
☒	☐	A description of any assumptions or corrections, such as tests of normality and adjustment for multiple comparisons
☐	☒	A full description of the statistical parameters including central tendency (e.g. means) or other basic estimates (e.g. regression coefficient) AND variation (e.g. standard deviation) or associated estimates of uncertainty (e.g. confidence intervals)
☐	☒	For null hypothesis testing, the test statistic (e.g. *F*, *t*, *r*) with confidence intervals, effect sizes, degrees of freedom and *P* value noted<br>*Give P values as exact values whenever suitable.*
☒	☐	For Bayesian analysis, information on the choice of priors and Markov chain Monte Carlo settings
☒	☐	For hierarchical and complex designs, identification of the appropriate level for tests and full reporting of outcomes
☒	☐	Estimates of effect sizes (e.g. Cohen's *d*, Pearson's *r*), indicating how they were calculated

Our web collection on statistics for biologists contains articles on many of the points above.

Software and code

Policy information about availability of computer code

Data collection	1.5 T clinical MRI instrument (iSpace Pro 1.5 T, Beijing Wandong Medical Technology Co., Ltd., Beijing, China); 7 T animal MRI instrument (Bruker BioSpec 70/20); 3 T human MRI instrument (GE SIGNA PET/MR); Microplate reader (Thermo, Varioskan Flash); Zetasizer (Malvern); ICP-MS (Thermo, ICAP-Qc); Transmission electron microscope (JEOL, JEM-2100); Gel Permeation Chromatography (GPC Waters 1515); 400 MHz Nuclear Magnetic Resonance Spectrometer (AVANCE III).
Data analysis	All data were analysed by commercial or open-source software: OriginPro (9.0 & 2019b); MatLab (R2019b); Graphpad Prism (v7.0 & v8.0); MestReNova (9.0.1.13254).

For manuscripts utilizing custom algorithms or software that are central to the research but not yet described in published literature, software must be made available to editors and reviewers. We strongly encourage code deposition in a community repository (e.g. GitHub). See the Nature Portfolio guidelines for submitting code & software for further information.

Data

Policy information about availability of data

All manuscripts must include a data availability statement. This statement should provide the following information, where applicable:

- Accession codes, unique identifiers, or web links for publicly available datasets
- A description of any restrictions on data availability
- For clinical datasets or third party data, please ensure that the statement adheres to our policy

All data supporting the findings described in this manuscript are available in the article, Supplementary Information, or Source data file (XLS). The MR imaging data

generated during the study is maintained by the College of Life Science and Technology, Beijing University of Chemical Technology, and will be shared for academic purposes on request (Dr. Yi Hou, houyi@iccas.ac.cn) for at least 5 years from date of publication. Source data are provided with this paper.

Human research participants

Policy information about studies involving human research participants and Sex and Gender in Research.

Reporting on sex and gender	n/a
Population characteristics	n/a
Recruitment	n/a
Ethics oversight	n/a

Note that full information on the approval of the study protocol must also be provided in the manuscript.

Field-specific reporting

Please select the one below that is the best fit for your research. If you are not sure, read the appropriate sections before making your selection.

☒ Life sciences ☐ Behavioural & social sciences ☐ Ecological, evolutionary & environmental sciences

For a reference copy of the document with all sections, see nature.com/documents/nr-reporting-summary-flat.pdf

Life sciences study design

All studies must disclose on these points even when the disclosure is negative.

Sample size	The sample sizes were set as at least 3 (n ≥ 3) in the imaging experiments and the biosafety evaluation of materials in vitro/in rodent animal models in vivo. Sample sizes were sufficient to show the same trends across replicates performed for each experiment.
Data exclusions	No data were excluded from the experiments.
Replication	At least triplicates were performed independently with similar results for each experiment. All experimental findings were reliably reproduced.
Randomization	The animals were randomly assigned to all experiments.
Blinding	In the biosafety evaluation experiments, researchers who performed data collection and analysis were blinded to group allocation and detailed materials information.<br>In the MRI studies, researchers who performed data collection, analysis and vascular identification were blinded to group allocation and detailed animals information.

Reporting for specific materials, systems and methods

We require information from authors about some types of materials, experimental systems and methods used in many studies. Here, indicate whether each material, system or method listed is relevant to your study. If you are not sure if a list item applies to your research, read the appropriate section before selecting a response.

Materials & experimental systems

n/a	Involved in the study	
☒	☐	Antibodies
☐	☒	Eukaryotic cell lines
☒	☐	Palaeontology and archaeology
☐	☒	Animals and other organisms
☒	☐	Clinical data
☒	☐	Dual use research of concern

Methods

n/a	Involved in the study	
☒	☐	ChIP-seq
☒	☐	Flow cytometry
☒	☐	MRI-based neuroimaging

Eukaryotic cell lines

Policy information about cell lines and Sex and Gender in Research

Cell line source(s)	Human umbilical vein endothelial cells (HUVECs) were purchased from American Type Culture Collection (ATCC).
Authentication	Cell lines authentication was performed by short tandem repeat DNA profiling and comparison with reference database.
Mycoplasma contamination	The cell line was tested negative for mycoplasma contamination by the mycoplasma detection kit (Yeasen Cat. No. 40611).
Commonly misidentified lines (See ICLAC register)	No, HUVECs are not listed in the database.

Animals and other research organisms

Policy information about studies involving animals; ARRIVE guidelines recommended for reporting animal research, and Sex and Gender in Research

Laboratory animals	Rodent animals including BALB/c mice (6-week-old), C57 mice (6-week-old) and Sprague Dawley (SD) rats (7-week-old) of the desired age were purchased from Vital River Animal Laboratories. The healthy bama swines (two-month and four-month, male) were purchased from Sichuan Greentech Bioscience Co., Ltd. Mice and rat were co-housed and maintained in SPF level animal room on a 12-hour light-dark cycle with free access to food and autoclaved water (22 ± 1°C, 50–60% humidity, 4 mice/cage, 2 rat/cage).
Wild animals	The study did not involve wild animals.
Reporting on sex	Discrimination of animal sex is not applicable to this study.
Field-collected samples	The study did not involve samples collected from the field.
Ethics oversight	All animal experiments were performed according to a protocol approved by the Peking University Institutional Animal Care and Use Committee (LA2019083).

Note that full information on the approval of the study protocol must also be provided in the manuscript.
